# Genomics of deletion 7 and 7q in myeloid neoplasm: from pathogenic culprits to potential synthetic lethal therapeutic targets

**DOI:** 10.1038/s41375-023-02003-x

**Published:** 2023-08-26

**Authors:** Minako Mori, Yasuo Kubota, Arda Durmaz, Carmelo Gurnari, Charnise Goodings, Vera Adema, Ben Ponvilawan, Waled S. Bahaj, Tariq Kewan, Thomas LaFramboise, Manja Meggendorfer, Claudia Haferlach, John Barnard, Marcin Wlodarski, Valeria Visconte, Torsten Haferlach, Jaroslaw P. Maciejewski

**Affiliations:** 1 Department of Translational Hematology and Oncology Research, Taussig Cancer Institute, Cleveland Clinic Foundation, Cleveland, OH USA; 2 Department of Hematology, National Hospital Organization Kyoto Medical Center, Kyoto, Japan; 3 Department of Biomedicine and Prevention, Ph.D. in Immunology, Molecular Medicine and Applied Biotechnology, University of Rome Tor Vergata, Rome, Italy; 4 Department of Hematology, St. Jude Children’s Research Hospital, Memphis, TN USA; 5 Department of Leukemia, The University of Texas MD Anderson Cancer Center, Houston, TX USA; 6 Department of Genetics and Genome Sciences, Case Western Reserve University School of Medicine, Cleveland, OH USA; 7 MLL Munich Leukemia Laboratory, Munich, Germany; 8 Department of Quantitative Health Sciences, Cleveland Clinic, Lerner Research Institute, Cleveland, OH USA

**Keywords:** Myelodysplastic syndrome, Acute myeloid leukaemia

## Abstract

Complete or partial deletions of chromosome 7 (-7/del7q) belong to the most frequent chromosomal abnormalities in myeloid neoplasm (MN) and are associated with a poor prognosis. The disease biology of -7/del7q and the genes responsible for the leukemogenic properties have not been completely elucidated. Chromosomal deletions may create clonal vulnerabilities due to haploinsufficient (HI) genes contained in the deleted regions. Therefore, HI genes are potential targets of synthetic lethal strategies. Through the most comprehensive multimodal analysis of more than 600 -7/del7q MN samples, we elucidated the disease biology and qualified a list of most consistently deleted and HI genes. Among them, 27 potentially synthetic lethal target genes were identified with the following properties: (i) unaffected genes by hemizygous/homozygous LOF mutations; (ii) prenatal lethality in knockout mice; and (iii) vulnerability of leukemia cells by CRISPR and shRNA knockout screens. In -7/del7q cells, we also identified 26 up or down-regulated genes mapping on other chromosomes as downstream pathways or compensation mechanisms. Our findings shed light on the pathogenesis of -7/del7q MNs, while 27 potential synthetic lethal target genes and 26 differential expressed genes allow for a therapeutic window of -7/del7q.

## Introduction

Complete or partial deletions of chromosome 7 (chr.7; -7/del7q) belong to the most frequent classes of chromosomal abnormalities found in myeloid neoplasm (MN) [1, 2]. -7/del7q can appear alone, or together with other chromosomal aberrations in the context of a complex karyotype (CK), in particular in therapy-related myelodysplastic syndrome (MDS) [3, 4]. Among chromosomal aberrations, -7/del7q stands out because of the associated poor prognosis, the high prevalence among pediatric MDS [2, 5, 6] and association with bone marrow failure (BMF) syndromes, including post-aplastic anemia (AA) MDS [2, 5–8].

Despite decades of studies, the various candidate genes responsible for the leukemogenic properties of -7/del7q have been proposed, but their role has not been completely elucidated, and no targeted therapies have been conceptualized [9–16]. Nevertheless, there are multiple, and not compatible hypotheses for the mechanisms by which -7/del7q may induce leukemic transformation. These theories implicate loss of heterozygosity (LOH), haploinsufficiency (HI) of tumor-suppressor genes (TSGs) and somatic rescue of suppressive germline variants located within the deleted regions. Deletions may create clonal vulnerabilities due to HI genes contained in the deleted regions, but not necessarily involved in leukemogenesis. Such genes may render clonal cells sensitive to direct synthetic lethal treatment strategies. In addition, HI of TSGs may trigger secondary effects, including compensatory up- or down-modulation of genes on other chromosomes, potentially constituting targets of indirect pharmacologic synthetic lethality [17, 18]. To date, these aspects of -7/del7q pathogenesis have been tested in a piecemeal manner, thereby precluding a precise and complete determination of its contribution to the development of MN.

Herein, we pursued a systematic plan to assess all of these alternative possibilities, and further exploit informative cases to pinpoint culprit genes and propose potential therapeutic strategies. For this purpose, we leveraged our large, well-characterized collection of -7/del7q patient samples to elucidate the full spectrum of the disease biology, including both inherited and sporadic cases harboring -7/del7q, taking advantage of multiple sources of genomic information, including whole-exome and genome sequencing, single-cell DNA-sequencing (scDNA-seq), and single-cell RNA sequencing (scRNA-seq) and bulk RNA sequencing (RNA-seq).

## Methods

### Patients and samples

A total of 8142 samples with MN were included in this study. We combined the data from Cleveland Clinic Foundation (CCF; *N* = 1667) and Munich Leukemia Laboratory (MLL; *N* = 4,573) with publicly available data (The Cancer Genome Atlas, the German-Austrian Study Group and the BEAT AML Master trial, and; *N* = 1902) (Table S1) [19–21]. All the CCF and MLL cases were diagnosed according to the 2016 WHO classification. Karyotype was confirmed by conventional metaphase cytogenetics (MC) and/or fluorescence in situ hybridization (FISH) analyses. Clinical and molecular data of the study were collected at the CCF or MLL, and retrieved from publicly available datasets. Informed consent was obtained from each patient for the collection of peripheral blood and bone marrow (BM) samples and the study was approved by the institutional review board at CC and other institutions in accordance with the Declaration of Helsinki.

### Genomic studies

For the CCF samples, 66, 92 and 1549 cases were performed by single nucleotide polymorphism array, whole-exome sequencing (WES) and targeted deep sequencing, respectively, as previously described [22–24]. MLL samples underwent whole genome sequencing (WGS) as previously reported [25–27]. The next-generation sequencing methods of publicly shared MN patients were previously described [19–21]. RNA-seq was performed on 49 MN cases with -7/del7q and 644 MN cases with diploid chr.7. Total RNA was extracted from BM samples and RNA-seq libraries were constructed and sequenced as previously described [22].

### Germline variants panel and filtering

Genomic DNA was isolated from purified CD3 positive cells from peripheral or BM blood mononuclear cells. Our germline targeted panel covered all of the exons of 163 targeted genes (Table S2). Detected variants were filtered and categorized as pathogenic or likely pathogenic variants as previously described [28].

### Clonal hierarchy

Clonality with -7/del7q was analyzed by copy number variation (CNV) from WES data in 23 samples and WGS data in 43 samples. CNV from WES or WGS data was calculated as previously described [22]. VAF of somatic variants was adjusted by zygosity and copy number. We determined dominant and secondary hits by comparing the -7/del7q clonality with adjusted variant allele frequency (VAF) of somatic variants. A cut-off at least 5% difference between clonality of del7 and VAF of somatic mutations was used to distinguish the dominant from second hits, whereas del7 and somatic mutations were referred to as co-dominant if the difference between those was less than 5%.

### Single cell DNA/RNA sequencing

Cryopreserved four and one BM sample was used for scDNA-seq and scRNA-seq analysis, respectively. Our custom scDNA-seq panels targeting 49 genes (Table S3) and CNV of chr5q, chr6p, chr7, and chr17 were designed and manufactured by Mission Bio. Detailed information is provided in supplementary methods.

### Haploinsufficiency expression analysis

RNA-seq data from 49 -7/del7q and 644 diploid MN samples were analyzed. Each gene expression level on chr.7 among 49 -7/del7q samples was adjusted to 100% clonality using the slope from the estimated linear model. The values of 644 diploid cases remained unchanged. Detailed information is presented in supplementary methods.

### Differential gene expression analysis

The Bayesian method by the linear models for microarray expression data (limma) package version 3.50.0 in R software was used for the normalization of genes and identification of differentially expressed genes between -7/del7q and NK MNs. The genes with log2 fold change (Log2FC) value > 1 or <-1, and -log(*q*-value)>5 were considered as significantly differentially expressed genes.

### Statistical analyses

Statistical analyses were performed by using GraphPad Prism (version 8) and R statistical software package (version4.1.2). Fisher’s exact test and Chi-square were used to compare categorical variables, while Mann-Whitney and Wilcoxon tests were used for pairwise continuous variables. The overall survival (OS) was defined from diagnosis to death or last follow-up and estimated using Kaplan–Meier method. Log rank test was used for comparison between groups.

All *P* values were considered statistically significant at *P* < 0.05. *P* values were corrected by employing the Benjamini–Hochberg method in detecting differentially expressed genes

## Results

### Clinical characteristics of -7/del7q MN patients

**Fig. 1:**
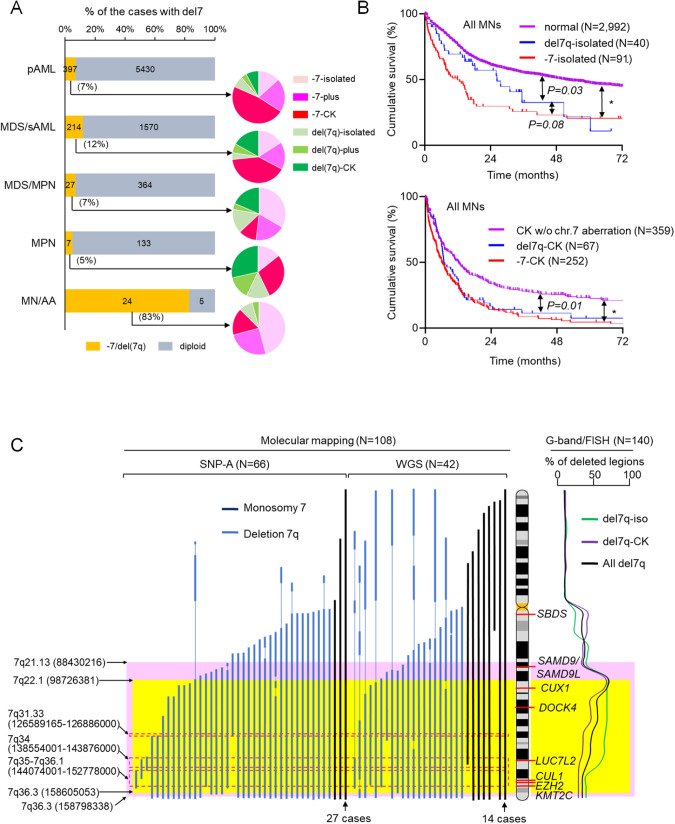
The disease distribution, prognosis, and common deleted regions (CDRs) of myeloid neoplasms with -7/del7q. **A** Frequency of -7/del7q per disease subsets among 8142 MNs. Pie charts showed distribution of karyotype (isolated, plus, and complex) in -7/del7q. **B** Survival outcomes of MN patients with normal karyotype, isolated del7q, and isolated -7 (upper) and survival curves of MN cases with complex karyotype (CK) without chromosome 7 (chr.7) aberration, del7q-CK, and -7-CK (lower). *P < 0.0001. **C** CDRs in del7q cases based on conventional metaphase cytogenetics (right) and molecular mapping of -7/del7q cases by SNP-A and WGS (left). Common haploinsufficient genes were shown on ideogram of the chr.7 FISH fluorescence in situ hybridization, MDS myelodysplastic syndrome, MDS/MPN myelodysplastic/myeloproliferative neoplasm, MN/AA myeloid neoplasms with previous aplastic anemia, MPN myeloproliferative neoplasms, pAML primary acute myeloid leukemia, sAML secondary acute myeloid leukemia, SNP-A single nucleotide polymorphism assay, WGS whole genome sequencing.

Among 8142 MN patients, we identified 501 with -7 and 144 with del7q (Table 1). In about half of the cases, -7/del7q was found in the context of CK, while 20–30% had -7/del7q as a sole (isolated) cytogenetic aberration. Accordingly, 7% of primary acute myeloid leukemia (pAML), 12% of MDS/secondary AML (MDS/sAML), 7% of MDS/myeloproliferative neoplasms (MDS/MPN), and 5% of MPN patients, harbored -7/del7q (Fig. 1A). Furthermore, up to 83% of MN with a previous history of AA carried -7/del7q, of which about 50% had isolated -7/del7q. Compared with MNs of normal karyotype (NK), MNs with isolated -7 or del7q had a poorer prognosis, while isolated -7 and del7q had a similar prognosis (*P* = 0.08) (Fig. 1B, upper). Among MNs with CK, patients with -7 or del7q showed significantly worse OS compared with CK-MNs without -7/del7q (Fig. 1B, lower). In addition, there was a significant prognostic difference between isolated and CK -7/del7q regardless of disease type (Supplementary Fig. 1).

**Table 1:** Baseline characteristics of patients with myeloid neoplasm in our study cohort.

	All MNs (*N* = 8,142)	Monosomy 7 (*N* = 501)	Del7q (*N* = 144)	*P*-value -7 vs del7q
Median age, years	68	69	68	0.49
Age < 40 year, %	6.2	5.3	5.6	0.89
Male, %	54.7	58.3	68.5	0.17
Diagnosis				
pAML, %	72	65	50	0.04
MDS, %	16	25	35	0.02
sAML, %	6	7	5	0.4
MDS/MPN, %	5	3	7	0.07
MPN, %	2	1	3	0.02
History of AA, %	0.4	4	2	0.24
Cytogenetics				
normal, %	53.5	-	-	-
del5/5q, %	5.4	22	30.1	0.07
isolated -7/7q, %	1.8	20	29.4	0.03
CK -7/del7q, %	7.7	56.6	52.4	0.5

*AA* aplastic anemia, *CK* complex karyotype, *MDS* myelodysplastic syndrome, *MDS/MPN* myelodysplastic/myeloproliferative neoplasm, *MNs* myeloid neoplasms, *MPN* myeloproliferative neoplasm, *pAML* primary acute myeloid leukemia, *sAML* secondary acute myeloid leukemia.

### Genomic features of -7/del7q MN

The commonly deleted regions (CDRs) were mapped to determine the most consistently affected genes. An analysis using metaphase cytogenetics (MC) showed the 7q22 region as the most commonly deleted region, consistent with previous reports [23, 29], but the 7q36 region was deleted in about 40% of the del7q (Fig. 1C). When looking at isolated del7q MN, the regions spanning q22-q34 were deleted in about 80% of the cases. For more precise mapping of CDRs, we analyzed the data of single nucleotide polymorphism array (SNP-A) and whole genome sequencing (WGS) among 50 cases with -7 and 58 cases with del7q. Nine cases with -7 detected by MC had incomplete loss of all regions on chr.7, but the majority had large deleted regions on 7q and 7p including the centromere. Among 108 cases with -7/del7q, 7q21.13-7q36.3 (88,430,216-158,798,338) and 7q22.1-7q36.3 (98,726,381-158,605,053) deletions were identified in >70% (pink area, Fig. 1C) and >80% of cases (yellow area, Fig. 1C), respectively. Regions corresponding to the bands 7q31.33 (126,589,165–126,886,000), 7q34 (126,589,165–126,886,000), and 7q35-7q36.1 (144,074,001-152,778,000) were deleted in >90% (area surrounded by red dotted line, Fig. 1C) of -7/del7q cases including *CUL1, EZH2, KMT2C*, and *LUC7L2* genes.

**Fig. 2:**
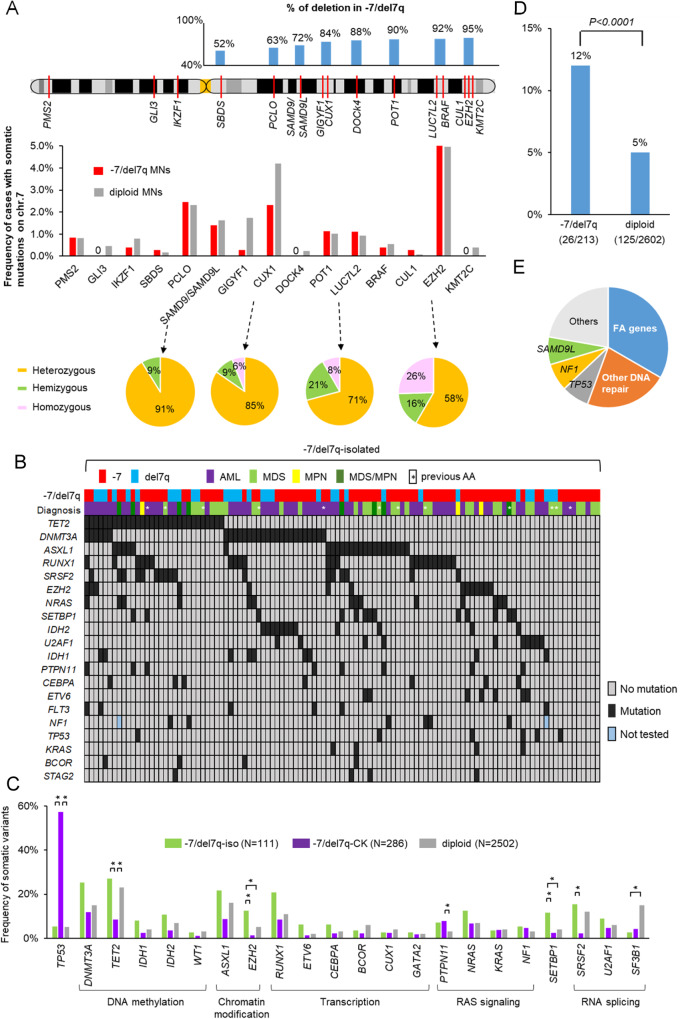
Landscape of coexisting somatic mutations and germline mutations in -7/del7 myeloid neoplasms. **A** Frequency of each deleted region in -7/del7q MNs according to molecular mapping analysis (upper), frequency of somatic mutations on chr.7 in -7/del7q (*N* = 518) and diploid (*N* = 2502) (middle), and distribution of zygosity configuration in each somatic mutation (lower). **B** Somatic mutational spectrum of isolated -7/del7q MN cases (*N* = 111). **C** Frequency of the somatic mutations was compared between isolated -7/del7q, CK -7/del7q, and diploid cases. *P < 0.001. **D** Prevalence of germline mutations in -7/del7q and diploid MNs. **E** Distribution of germline mutations detected in the 26 MNs with -7/del7q.

We then focused on the molecular profiles on chr.7 through analyzing the frequency of somatic mutations in MN cases with -7/del7q and intact chr.7 (diploid) (Table S4). In total, 16 genes (*BRAF, CUL1, CUX1, EZH2, DOCK4, GIGYF, GLI3, IKZF1, KMT2C, LUC7L2, PCLO, PMS2, POT1, SAMD9, SAMD9L, and SBDS*) on chr.7 were found to be recurrently mutated (Fig. 2A). Among them, *EZH2* mutations were the most common in both -7/del7q and diploid MNs (25/518 [5%] in -7/del7 and 124/2502 [5%] in diploid). Specifically, in *EZH2*-mutants (*n* = 149), we found 24 cases (16%) with hemizygous (one allele of chr.7 containing *EZH2* was deleted and another allele had a *EZH2* mutation) in -7/del7q MNs and 38 cases (26%) with homozygous configuration in diploid MNs. Notably, 13 truncations and 11 pathogenic missense mutations mostly in the SET domain were found in patients with *EZH2* hemizygous configuration (Table S5). *CUX1* mutations tended to be less frequent in -7/del7q (12/518, 2.3%) than in diploid cases (105/2502, 4.2%). Among 117 cases with *CUX1* mutants, 11 cases (9%) were hemizygous in -7/del7q and 7 (6%) were homozygous in diploid (Table S6). Several cases with *EZH2* or *CUX1* biallelic LOF mutations were found in both -7/del7q and diploid MNs. We also identified somatic mutations in *PCLO* (9/357 [2.5%] vs. 43/1849 [2.3%] detected in diploid cases), *SAMD9/SAMD9L* (5/357 [1.4%] vs. 30/1849 [1.6%] in diploid), and *LUC7L2* (5/451 [1.1%] vs. 19/2502 [0.8%] in diploid). Conversely, somatic hits were rare in *CUL1, DOCK4, KMT2C*, and *SBDS* (Fig. 2A).

When we analyzed the frequency of the somatic mutations on all chromosomes in both -7/del7q and diploid cases, in isolated -7/del7q, the most recurrent mutated genes were *TET2* (27%), followed by *DNMT3A* (25%), *ASXL1* (22%), *RUNX1* (21%), *SRSF2* (15%), and *EZH2* (13%) (Fig. 2B and Supplementary file). Isolated -7/del7q showed significantly higher frequencies of mutations in *EZH2* and *SETBP1*, but less *SF3B1* mutations compared with diploid cases (Fig. 2C), and no difference was found between isolated -7 and del7q (Supplementary Fig. 2A). In -7/del7q cases with one additional chromosomal aberration (-7/del7q-plus), inv(3)(q21q26) or t(3;3)(q21;q26) were found in 21%, while del5q, del12p and del20q were observed in only 6% of cases each (Supplementary Fig. 2B, C). In -7/del7q-CK, the frequency of *TP53* mutations was high (57%; Supplementary Figs. 2C and 3), and co-occurring cytogenetic aberrations were del5q (57%), del17p (26%), del12p (17%), and del20q (10%).

Germline mutations (panel of selected 163 cancer-associated genes, Table S2) were detected more often in -7/del7q as compared to diploid MN (12% *vs*. 5%, *P* < 0.0001; Fig. 2D). In particular, heterozygous Fanconi anemia (FA) or other DNA repair associated gene mutations constituted about 50% of all germline lesions (Fig. 2E, Table S7). Although a high prevalence of germline *SAMD9/9L* or *GATA2* mutations were reported in pediatric MDS with -7 [16], in our cohort of adult patients, *SAMD9L* germline mutations were identified in only 2 cases while no *SAMD9* nor *GATA2* germline alterations were found.

### Clonal hierarchy of -7/del7q MN

**Fig. 3:**
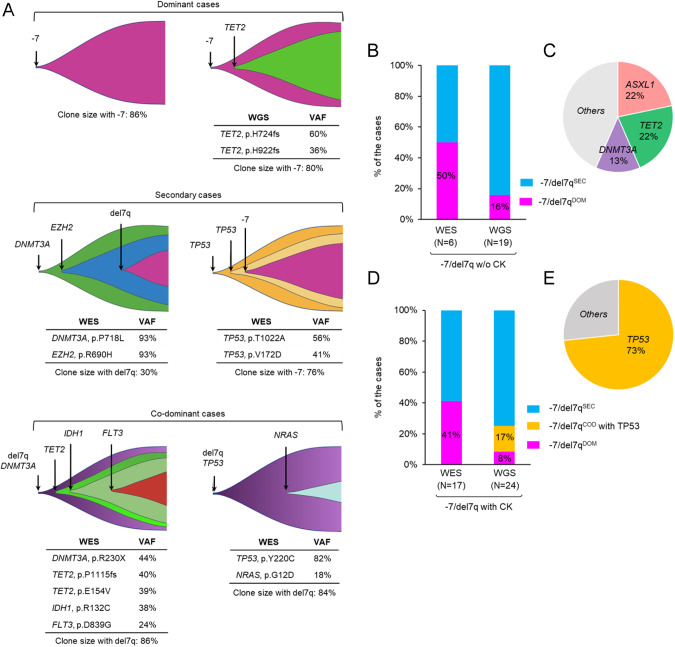
Clonal architecture of myeloid neoplasms with -7/del7q. **A** Exemplary cases of -7/del7q dominant (upper), secondary (middle), and co-dominant with somatic variants (lower). Reconstruction of the clonal hierarchy was analyzed using an allelic imbalance for WES samples and CNV analyses for WGS. Figures were created with BioRender.com. **B** Distribution of dominant or secondary -7/del7q hit in -7/del7q MN cases without complex karyotype (CK). **C** Dominant somatic mutational distribution in secondary -7/del7q cases without CK. **D** Distribution of dominant, secondary, or co-dominant -7/del7q hit in CK -7/del7. **E** *TP53* mutation was dominant hit in most of -7/del7q cases with CK.

To define the initial molecular events in the pathogenesis of -7/del7q, we first analyzed the clonal hierarchy of -7/del7q MN using the VAF-method [22]. Clonal sizes were calculated using WES data in 23 BM samples and WGS data in 43 BM samples. Clonal succession with regard to mutational events was resolved in 66 cases assigning -7/del7q as dominant (-7/del7q^DOM^), secondary (-7/del7q^SEC^), and co-dominant when distinction was not arithmetically possible (-7/del7q^COD^; Fig. 3A). A VAF of 50% means that essentially all diploid cells carry a mutated copy in their one allele. Among 25 cases with isolated or plus -7/del7q, 6 were -7/del7q^DOM^ and 19 were -7/del7q^SEC^ (Fig. 3B). In 19 isolated/plus -7/del7q^SEC^ cases, *ASXL1* and *TET2* (22% each) were the most common dominant somatic hits (Fig. 3C), whereas in -7/del7q-CK MN, 73% had dominant/co-dominant *TP53* mutations (Fig. 3D, E).

**Fig. 4:**
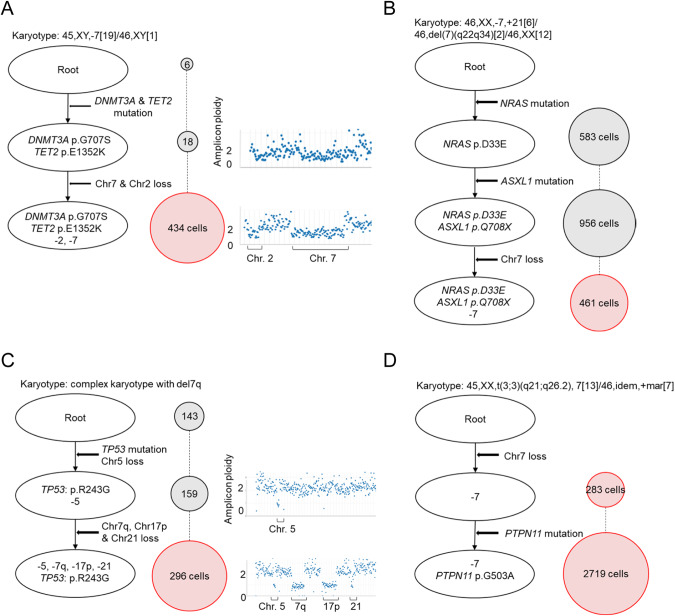
Single-cell DNA-sequencing resolving the clonal architecture of -7/del7q. (**A**–**D**) Four cases with myeloid neoplasm carrying -7/del7q were subjected to single-cell DNA-sequencing. The number of cells with each mutation or copy number abnormality are shown in the circle on the right. The cell populations with -7/del7q are shown in pink-colored circle.

We also applied scDNA-seq in 4 cases harboring -7/del7q. One patient with AML with isolated -7 harbored three independent clones, including native state cells (wild-type clone), a clone with *DNMT3A* and *TET2* mutations and an additional clone with the above-mentioned somatic mutations and haploid chr.7 (Fig. 4A), suggesting that loss of chr.7 was a secondary event. A second patient with MDS showed four independent clones, including native state cells, one clone with only one *NRAS* somatic mutation, one clone with *NRAS* and *ASXL1*, and one clone with *NRAS*, *ASXL1*, and -7, suggesting a subclonal loss of chr.7 (Fig. 4B). Likewise, a patient with CK-del7q had a dominant *TP53* mutation with loss of chr.5 and subclonal del7q loss (Fig. 4C). In contrast, another AML case with -7 and t(3;3)(q21;q26) showed the loss of chr.7 in a dominant configuration (Fig. 4D). Although -7/del7q has been thought to be a primary event for the development of MN especially in isolated -7/del7q cases [9], both our bulk sequencing and scDNA-seq analyses indicated that -7/del7q is not an obligatory primary event for leukemogenesis.

### Haploinsufficient genes on chromosome 7

**Fig. 5:**
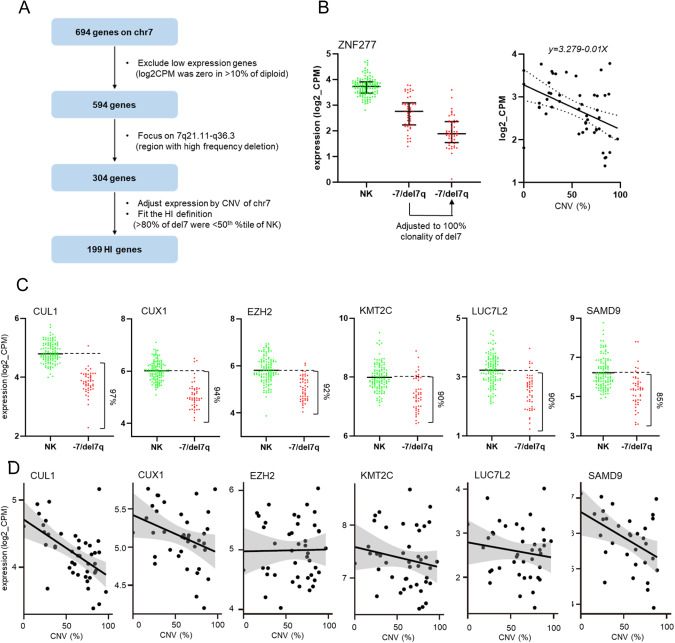
Haploinsufficient (HI) gene analysis of -7/del7q cases. **A** Flowchart for selecting HI genes using mRNA-sequencing data of 49 -7/del7q and 120 normal karyotype (NK) cases **B** ZNF277 was shown as an example of selected HI genes. ZNF277 expression levels of NK cases, -7/del7q before adjustment, and -7/del7q after adjustment were shown (left). Each expression level of -7/del7q cases was adjusted to 100% clonality using the slopes from estimated linear model (right). **C** Six common HI genes fitted our HI definition. **D** Correlation between copy number variation (CNV) and gene expression in 6 well-known HI genes.

To identify HI genes on chr.7, we analyzed RNA-seq data of 49 -7/del7q and 120 NK MNs (without chr.7 microdeletions). Of the initial 694 genes, 100 genes were excluded given their very low expression levels in >10% of diploid samples (Fig. 5A). We then focused on 304 genes located in 7q21-7q36 region, selected because of its high frequency of deletion (>70% in -7/del7q cases, Fig. 1C). Expression levels of the 304 genes among -7/del7q samples were adjusted to 100% clonality using the slopes from estimated linear model (-7/del7q clonality inversely correlated with expression levels, Fig. 5B). This strategy removed experimental noise stemming from the variable content of clonal -7/del7q cells. We then defined expression of this more restricted selection of -7/del7q genes to be HI, if the levels were <50th %tile of normal levels in >80% of the -7/del7q cases (Table S8) and thereby we obtained 199 genes, including *e.g*., CUL1, CUX1, EZH2, KMT2C, LUC7L2, and SAMD9 (Fig. 5C, D). However, other well-known HI genes like SAMD9L and DOCK4 [30] were excluded because only 77% and 71% of the cases had expression levels <50th %tile as compared to NK (Table S8). SBDS was also excluded because it was located on 7q11.21 and deleted in only 57% of -7/del7q cases.

**Fig. 6:**
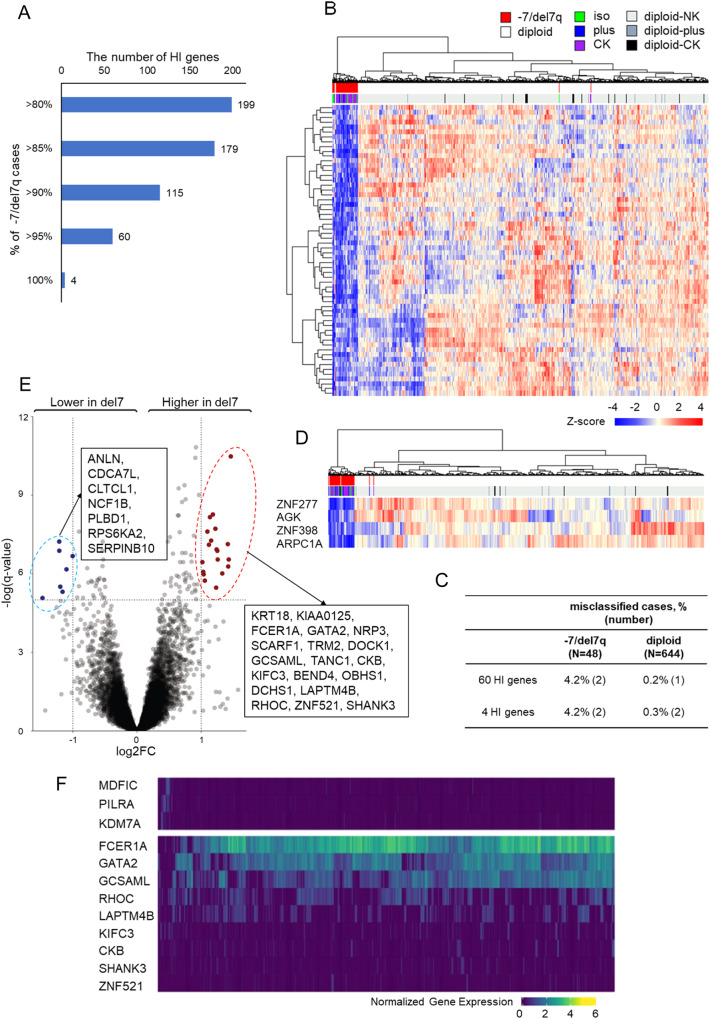
Haploinsufficient (HI) genes signature and differentially expression genes (DEGs) of -7/del7q myeloid neoplasms. **A** The number of HI genes using each HI definition: more than 80%, 85%, 90%, 95%, and 100% of -7/del7q had gene expression levels less than 50th percentile of those of NK cases. **B** Heatmap using unsupervised clustering of -7/del7q and diploid cases based on the 60 HI genes signature. **C** Frequency of the misclassified -7/del7q or diploid cases by 60 and 4 genes signature. **D** Heatmap for 4 genes minimal signature of -7/del7q. **E** DEGs on other chromosomes between -7/del7q and NK. **F** Heatmap using unsupervised clustering of BM cells from a patient with AML carrying monosomy 7 by our del7 genes signature based on single-cell RNA sequencing data.

When stringent criteria of HI, <50th %tile of the NK cases in more than 95% of the -7/del7q cases were applied, 60/199 HI genes fulfilled the criteria (Fig. 6A). Using these 60 HI genes signature, we performed an unsupervised clustering (Fig. 6B), which was able to distinguish -7/del7q from diploid cases with only two of -7/del7q and one diploid case being misclassified (Fig. 6C). The minimal gene signature included AGK, ARPC1A, ZNF277, and ZNF398, and showed an error rate for clustering within -7/del7q and diploid of 4.2% and 0.3%, respectively (Fig. 6D).

We also compared the expression of genes located outside of our defined CDRs on chr.7 and on other chr.7 regions between -7/del7q and NK cases (Table S9). Overall, 19 genes were significantly up-regulated in -7/del7q compared with NK, while 7 genes were significantly down-regulated (Fig. 6E). These genes included one TSG (RPS6KA2) and two oncogenes (LAPTM4B, RHOC).

We then analyzed the 60 down-regulated gene expression signature of -7/del7q and indirectly up-modulated 19 genes on other chromosomes at a single-cell level. A filtering algorithm to remove scRNA-seq dropouts led to the selection of three down and nine up-regulated genes as -7/del7q signature (Supplementary Fig. 4A). When these 12 genes were analyzed in one sample with AML carrying -7, we identified a small population of likely wild-type cells characterized by high mRNA expression levels of MDFIC, PILRA and KDM7A and majority of remaining cell population with -7/del7q signature (Fig. 6F). We then investigated the -7/del7q gene signature on CD33, CD117 or CD14 expressing cells. We found that our del7 gene signature was mostly enriched in CD33 and CD117-positive cells (Supplementary Fig. 4B–D), while the wild-type cells were overrepresented among CD14^+^ cells (Supplementary Fig. 4E).

### Synthetic lethal targets for -7/del7q MN

**Fig. 7:**
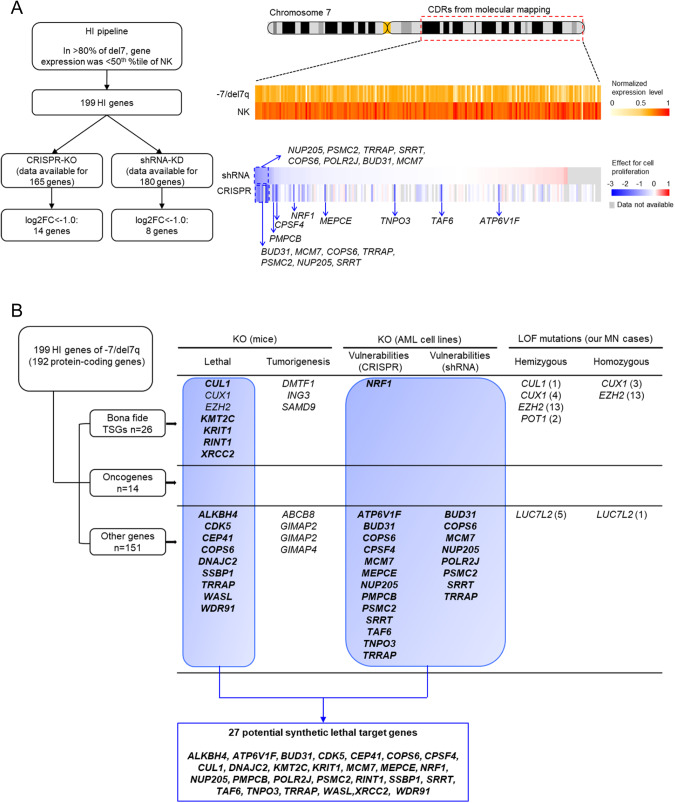
Multiple haploinsufficient (HI) genes involved in leukemia cell survival and potential synthetic lethal target genes for -7/del7q myeloid neoplasm. **A** Flowchart (left) for selecting the genes, CRISPR/shRNA knockout (KO) of which showed significant cell growth inhibition or a trend towards cell proliferation. Graphic (right) depicting the differentially gene expression levels between -7/del7q and NK myeloid neoplasms of the 199 HI genes in the CDRs identified by molecular mapping and the effect of shRNA or CRISPR KO of the 199 HI genes on cell proliferation from DepMap public database (https://depmap.org/portal/). **B** Summary of 199 HI genes based on lethality in KO mice collected from literature review, vulnerability of leukemia cells by CRISPR/shRNA KO screens from DepMap database, and the presence of cases affected by hemizygous or homozygous LOF mutations in our cohort. The numbers in brackets indicate the number of cases with each hemizygous or homozygous mutation in our cohort.

Among 192 HI protein coding genes, 26 were commonly recognized as TSGs (https://bioinfo.uth.edu/TSGene/), whereas 15 were known oncogenes (https://oncovar.org/welcome/index). In the remaining 151 genes, various potential TSGs, related to chromatin regulation, DNA replication or DNA damage response, and essential genes for cell-survival related to actin network, Ubl conjugation pathway, RNA splicing or ZNF-finger proteins, were identified. The 199 HI genes were further investigated for their functional effects and possible utility as potential targets for therapy in -7/del7q MN. For this purpose, we used the cancer dependency map (DepMap) (https://depmap.org/portal/) containing results of genome-wide CRISPR-Cas9 or shRNA screens [31, 32]. CRISPR knockout (KO) screen data was available for 165 of 199 HI genes in 26 different AML cell lines (Fig. 7A). KO of 14/164 genes showed significant cell growth inhibition. From the shRNA screen data for 180/199 genes in 32 AML cell lines, KO of 8 genes showed significantly reduced cell proliferation. A total of 15 genes (*ATP6V1F, BUD31, COPS6, CPSF4, NUP205, MCM7, MEPCE, NRF1, POLR2J, PMPCB, PSMC2, SRRT, TAF6, TNPO3, TRRAP*) for which, CRISPR/shRNA KO demonstrated a significantly reduced leukemic cell proliferation and suggested that they are essential for leukemia cell survival and possible synthetic lethal targets for -7/del7q MN (Fig. 7A).

Finally, we performed an integrative analysis of HI genes considering CRISPR/shRNA screen results, lethality in KO mice collected from literature review and inclusion of genes affected by hemizygous or homozygous LOF mutations in our study cohort (Fig. 7B). Previous studies showed embryonic or perinatal lethality in KO mice for 16 genes (*ALKBH4, CDK5, CEP41, COPS6, CUL1, CUX1, DNAJC2, EZH2, KMT2C, KRIT1, SSBP1, RINT1, TRRAP, WASL, WDR9*1 and *XRCC2*) (Table S10). While several cases of *CUX1, EZH2*, and *LUC7L2* hemizygous and homozygous LOF mutations were found in our cohort, the presence of natural KO compatible with survival makes them less likely candidates of synthetic lethality. In addition, OS was shorter in patients with *EZH2* hemizygous or homozygous mutations than in those with heterozygous configuration arguing against its use as a synthetic lethal target (Supplementary Fig. 5). Overall, we identified a total of 27 potentially synthetic lethal target genes out of 199 HI genes (Fig. 7B).

## Discussion

In this study, we performed a comprehensive integrative analysis of -7/del7q in a large cohort of patients with MN. Previous evidence on this topic utilized either limited number of patients, specific clinical phenotypes or were restricted to one or a few analytic techniques [7, 23, 33, 34]. Considering that the genetic features of -7/del7q are diverse when occurring as an isolated lesion or in the context of CK, having at our disposal the power of a large cohort of MN cases, we were able to precisely discern clinical differences, including survival, morphologic links and crucial features of key pathogenic associations. First, cognizant of the different cytogenetic risk classification of the revised IPSS, with -7 classified as poor and del7q belonging to the intermediate-risk group [4], we demonstrated in a larger -7/del7q MN cohort that there was no significant difference in prognosis between -7 and del7q in either isolated or CK MN cases.　Consistent with these results, the somatic mutational landscape did not differ between isolated -7 vs. del7q and between CK -7 vs. del7q. In addition, CK with -7/del7q had a significantly worse prognosis than CK without -7/del7q, suggesting that the deletion of 7q is an extremely unfavorable prognostic factor among CK-MNs.

Second, given the genomic complexity of MN carrying aberration of chr.7, we performed a multimodal clonal hierarchy analysis and revealed that -7/del7q could be both a founder as well as a subclonal lesion to specific pattern of mutations. Indeed, the frequently mutated genes such as *TET2, ASXL1*, and *DNMT3A* were founder in >50% of isolated -7/del7q and *TP53* in >70% of CK cases. These findings pinpoint how intricate dynamics beyond the mere presence of -7/del7q cooperate in shaping the clonal hierarchy and resultant trajectories of individual patients, suggesting that that chr. 7 aberrations may be secondary to molecular lesions [35].

The two most pressing research questions revolving around -7/del7q have been: (1) the identification of the culprit genes most faithfully phenocopying the pro-leukemogenic effects of the deletion, and (2) the possible existence of genes affected by the -7/del7q, which are essential for cell survival, and thereby might be synthetically lethal if targeted by drugs [9, 35, 36].

To advance along the first question, we have deployed a new bioinformatic strategy to improve the analysis of HI gene expression related to -7/del7q CDR. Our approach involved innovative determination of a linear inverse relationship between mRNA levels and clonal size with -7/del7q and normalization of the mRNA expression to 100% -7/del7q clonality, thereby making the expression of HI genes comparable between patients with otherwise varying fractions of clonal cells. Genes deleted in more than 70% of -7/del7q cases by WGS or SNP-A-based mapping were compared in their expression to NK cases without any microdeletion on chr.7; expression <50th %tile of diploid controls in >80% of -7/del7q patients were identified with 199 HI genes. These genes included not only already known HI genes (*CUL1, CUX1, EZH2, KMT2C, LUC7L2, SAMD9*) but also various bona fide TSGs (*KRIT1, RINT1, XRCC2, NRF1*) and a variety of other potential TSGs involved in DNA damage response, DNA replication and chromatin regulator [9, 37, 38]. Through unsupervised clustering and scRNA-seq analyses we could confirm that these genes were truly HI genes in -7/del7q MN. One could speculate that mechanisms of impaired DNA damage repair may be involved in the ontogenesis of MN with -7/del7q, consistent with their enriched frequencies in context of prior genotoxic exposure (e.g., therapy-related MN) or hyper-expansion of a stressed BM (e.g., post-AA, post-gene therapy) [39–41].

Concerning the second pressing aspect of -7/del7q, we identified 199 HI genes which were filtered using the following criteria: (1) unaffected genes by hemizygous/homozygous LOF mutations (2) prenatal lethality in KO mice from literature review and (3) vulnerability of leukemic cells by CRISPR or shRNA KO screens using the DepMap database. As a result, we narrowed down a total of 27 potentially synthetic lethal target genes. Among the 27 genes, *COPS6* and *TRRAP* have been shown to be embryonic lethal in KO mice [42, 43] and to be significantly vulnerable in leukemic cells with CRISPR or shRNA KO. Similarly, *CUL1*, a well-known HI gene associated with -7/del7q has been recognized as a promising candidate because (i) its expression resulted ubiquitously HI in almost all -7/del7q cases, (ii) it showed an ideal negative slope in clonal size and gene expression, (iii) few cases had biallelic LOF mutations, and (iv) it was previously associated with embryonic lethality in KO mice [44].

Although *CUX1* and *EZH2* were both HI in more than 90% of -7/del7q cases in our study and lethality of KO mice for these genes has been reported [45, 46], several cases with hemizygous/homozygous LOF mutations were found in our cohort and MNs with biallelic LOF *EZH2* or *CUX1* mutations appeared to exhibit an aggressive phenotype [47]. Altogether these findings suggest that *CUX1* and *EZH2* less likely serve as synthetic lethal targets.

Finally, we also identified 26 consistently up or down-regulated genes mapping on other chromosomes in -7/del7q MN cases. Of note is the two oncogenes (LAPTM4B and RHOC) up-regulated among the 26 genes have been proposed as potential therapeutic targets [48, 49].

In conclusion, we compiled a large genomic dataset of -7/del7q MNs, showing precise context-specific genomic features and identifying a transcriptomic signature encompassing a selection of consistently HI genes on 7q and genes outside chr.7 differentially expressed in -7del7q. These findings shed further light on the pathogenesis of -7/del7q MNs, while supplying a selection of new potential therapeutic approaches, subject of future knockout and knockdown studies.

### Supplementary information

Supplementary information

Supplementary Table 4

Supplementary Table 9

## Data Availability

Scripts for data processing and analyses of bulk and single-cell RNA-seq are available at http://github.com/ysokbt/monosomy7. For additional information, please contact the corresponding author: maciejj@ccf.org.
