# Supplementary material for: Genomics of deletion 7 and 7q in myeloid neoplasm: from pathogenic culprits to potential synthetic lethal therapeutic targets
Source: Leukemia. 2023 Aug 26;37(10):2082–93. doi: 10.1038/s41375-023-02003-x (PMC10539177; doi:10.1038/s41375-023-02003-x)
Supplement: Supplementary file 1 — Supplementary information [file 41375_2023_2003_MOESM1_ESM.docx]

**Supplemental information for**

**Genomics of deletion 7 and 7q: from pathogenic culprits to potential synthetic lethal therapeutic targets**

Minako Mori, Yasuo Kubota, Arda Durmaz, Carmelo Gurnari, Charnise Goodings, Vera Adema, Ben Ponvilawan, Waled S. Bahaj, Tariq Kewan, Thomas LaFramboise, Manja Meggendorfer, Claudia Haferlach, John Barnard, Marcin Wlodarski, Valeria Visconte, Torsten Haferlach, Jaroslaw P. Maciejewski

Supplemental information for this manuscript includes the following:

Supplemental methods

Supplemental References

Supplemental Figure1-5

Supplemental Table1, 2, 3, 5, 6, 7, 8, 10

(Supplemental Table 4 and 9 were attached separately by excel files)

**Supplemental methods**

*Single cell DNA sequencing*

Cryopreserved four BM samples were used for scDNA-seq analysis. As previously reported, scDNA-seq was performed using the Mission Bio Tapestri　(South San Francisco, CA) platform (1). The custom scDNA-seq panels targeting 49 genes (Table S8) and CNV of chr5q, chr6p, chr7, and chr17 were designed and manufactured by Mission Bio.　Variants and CNV analyses were conducted using Tapestri Insights platform version 2.2. CNV analysis was done using Mosaic (Mission Bio). The ratio of allele dropout in our four samples was 12.1% (Figure 4A), 23.9% (Figure 4B), 23.4% (Figure 4C), and 0% (Figure 4D).

*Single cell RNA sequencing*

A cryopreserved BM sample with AML carrying -7 was processed using the 10x Genomics platform and the chromium single cell 3' v3.1 chemistry. Library preparation was done using standard manufacturer’s protocols. Demultiplexing, barcode filtering, alignment and read counting was done using Cellranger software version 4.0 with default parameters. Approximately 5700 cells were kept after quality control. Further, filtering was done via python scanpy package where genes with i) less than 100 cells expressed, ii) cells with less than 800 genes expressed, iii) cells with more than 10% reads mapping to mitochondrial genes and iv) cells with less than 5000 total reads are removed. Finally, genes with normalized dispersion estimates less than 0.1 were removed and expression levels were library size-normalized and log-transformed.

60 genes identified as HI genes signature in -7/del7q cases were investigated in single-cell dataset matching only 3 genes based on the preprocessing of scRNA-seq data. In addition to 60 genes, another set of 19 auxiliary genes up-regulated in -7/del7q cases were included to increase sensitivity as well out of which only 12 matched with filtered scRNA-seq data. In order to visualize the expression profiles of individual cells, we generated a heatmap using ComplexHeatmap package in R. Furthermore, to emphasize the small possible wild-type cell population, we generated a low-dimensional representation of 15 genes in total using UMAP with parameters where the color gradient represents the average expression of 12 auxiliary genes; number of neighbors set to 100, minimum distance set to 0.5 and euclidean distance as the metric for nearest neighbor graph.

*Haploinsufficiency expression analysis*

Each gene expression level on chr.7 among 49 -7/del7q samples was adjusted to 100% clonality using the slope from the estimated linear model with log2(CPM+1) to remove the clonal heterogeneity based on the clonality levels and expression levels of genes in chr.7 in -7/del7q cases (2). The values of 644 diploid cases remained unchanged. HI genes were filtered using the following criteria: (i) excluding genes that were not expressed in more than 10% of the diploid samples: (ii) excluding genes with lower than the average expression levels of diploids in less than 80% of -7/del7q; (iii) excluding genes on chromosome 7p and 7q11. HI genes and the minimal signature genes were normalized using z-score normalization. Heatmaps were generated using the R package heatmap version1.0.12. Pearson correlation was used for the clustering method.

References

1. Awada, H. [Kerr](https://pubmed.ncbi.nlm.nih.gov/?sort=date&term=Kerr+CM&cauthor_id=33603144) CM , [Durmaz](https://pubmed.ncbi.nlm.nih.gov/?sort=date&term=Durmaz+A&cauthor_id=33603144) A, et al*.* Clonal trajectories and cellular dynamics of myeloid neoplasms with SF3B1 mutations. *Leukemia.* 2021*;*35(11):3324-3328
2. Adema V.  [Palomo](https://pubmed.ncbi.nlm.nih.gov/?sort=date&term=Palomo+L&cauthor_id=35617825) L , [Walter](https://pubmed.ncbi.nlm.nih.gov/?sort=date&term=Walter+W&cauthor_id=35617825)  W, et al. Pathophysiologic and clinical implications of molecular profiles resultant from deletion 5q. *EBioMedicine*. 2022;80:104059


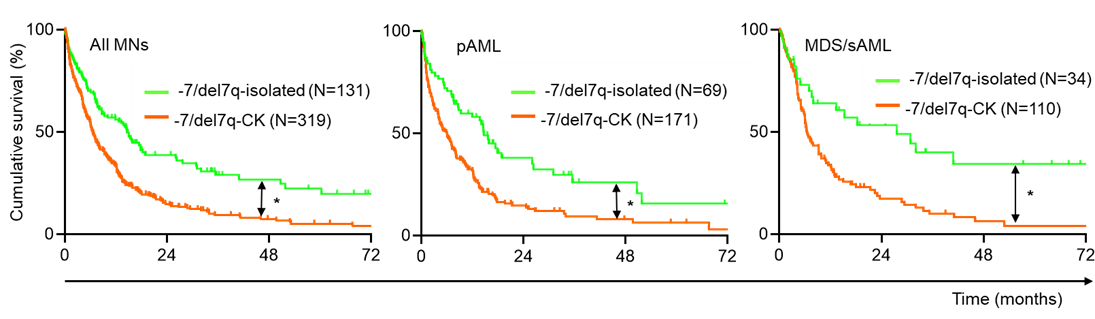


**Supplementary Figure 1**

Differences in overall survival between isolated -7/del7q and CK -7/del7q for each disease


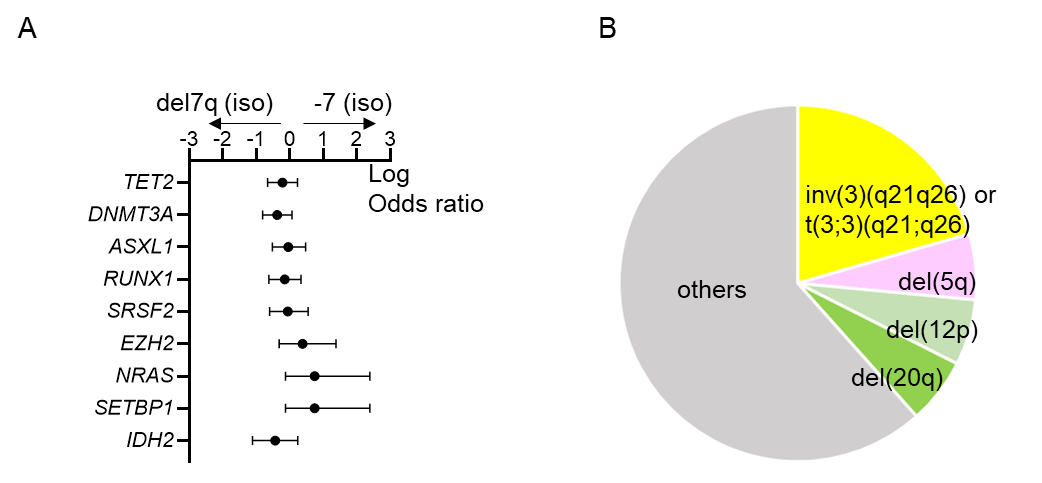


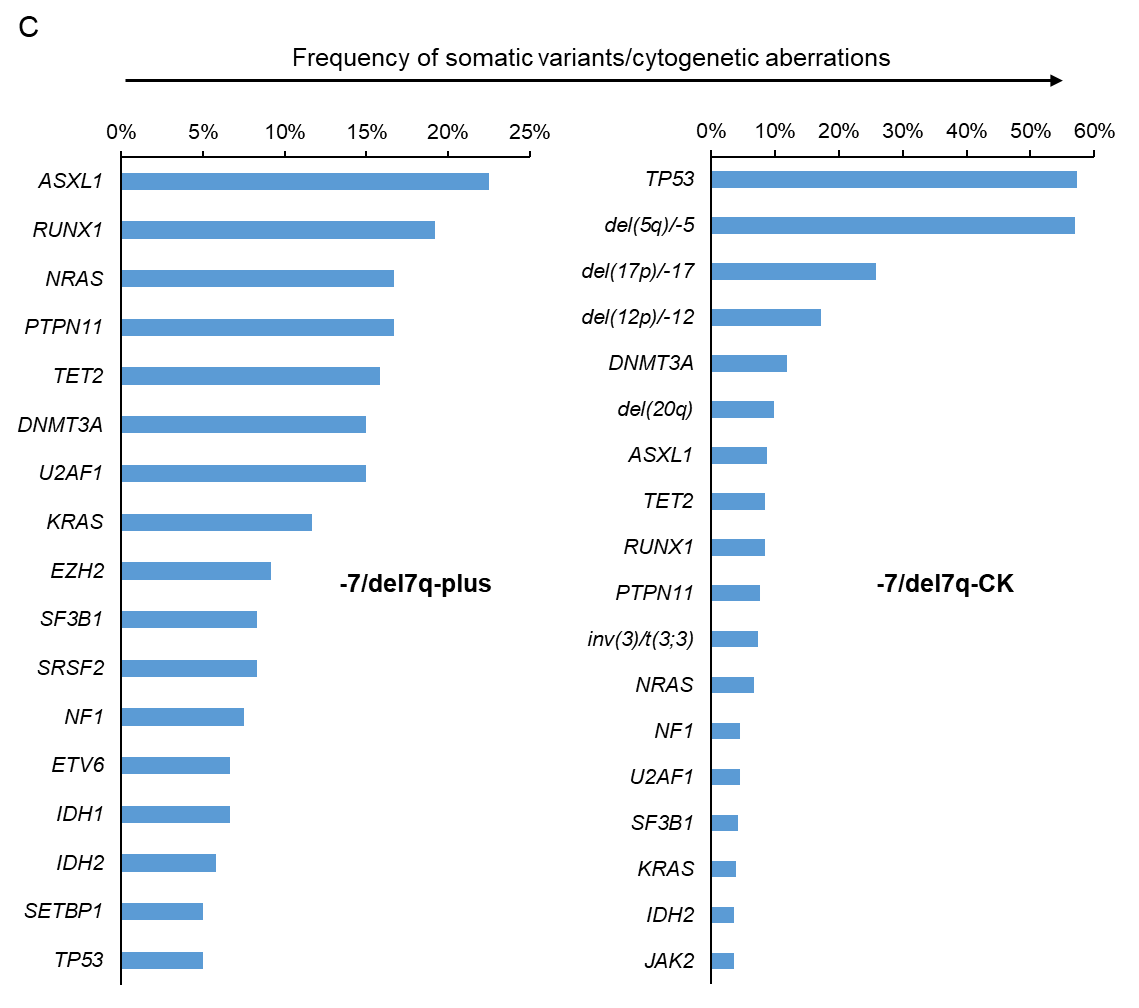


**Supplementary Figure 2: Co-existing somatic mutations and chromosomal abnormalities in -7/del7q myeloid neoplasms (MNs)**

1. The odds ratio representing the strength of the association of isolated -7 or isolated del7q with high frequent somatic mutations.
2. Distribution of chromosomal aberrations in -7/del7q cases with one chromosomal aberration (plus)
3. Percentage of the co-associated mutations or cytogenetic aberrations in -7/del7q-plus MNs (Left) or -7/del7q with complex karyotype (CK) (Right).


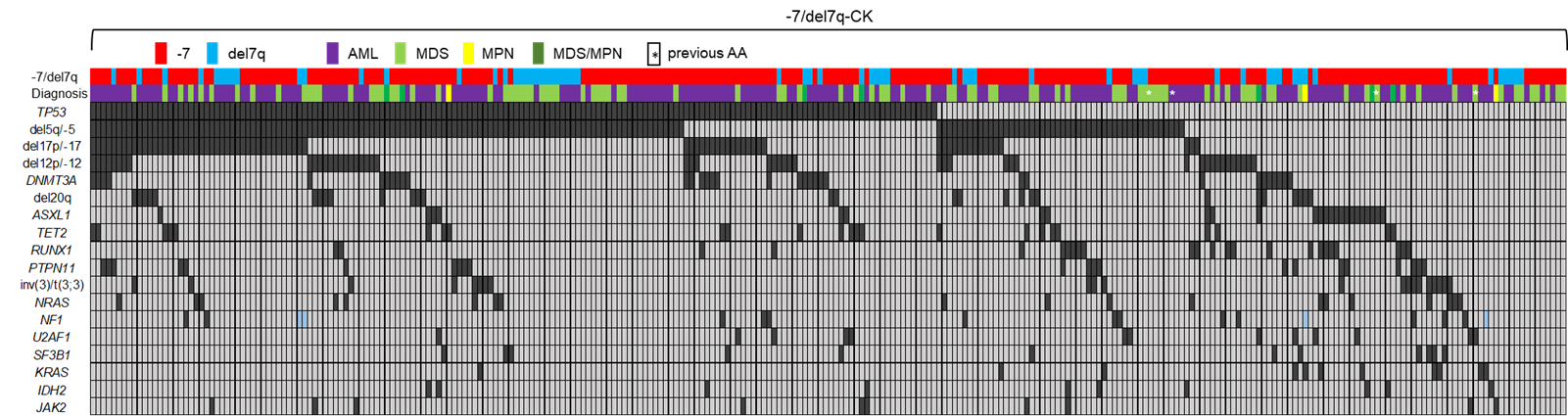


**Supplementary Figure 3: Landscape of the associated mutations and chromosomal abnormalities in -7/del7q with complex karyotype (CK)**

AA, aplastic anemia; AML, acute myeloid leukemia; MDS, myelodysplastic syndrome; MPN, myeloproliferative neoplasm; MDS/MPN, myelodysplastic/myeloproliferative neoplasm


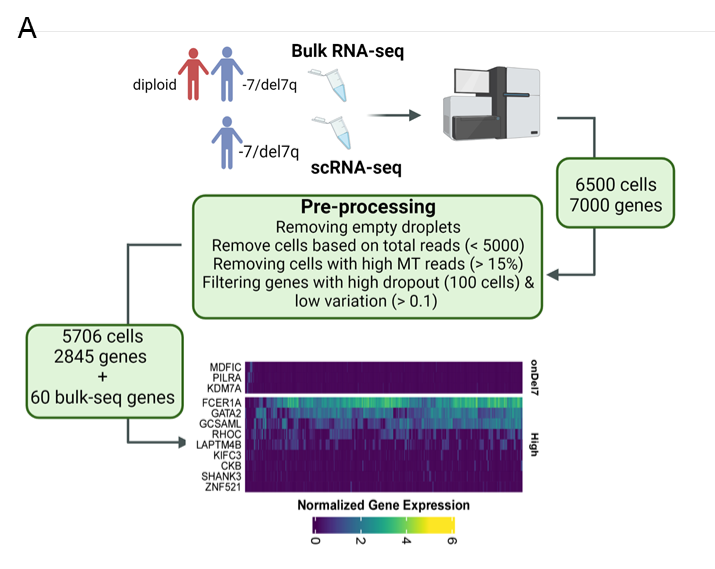

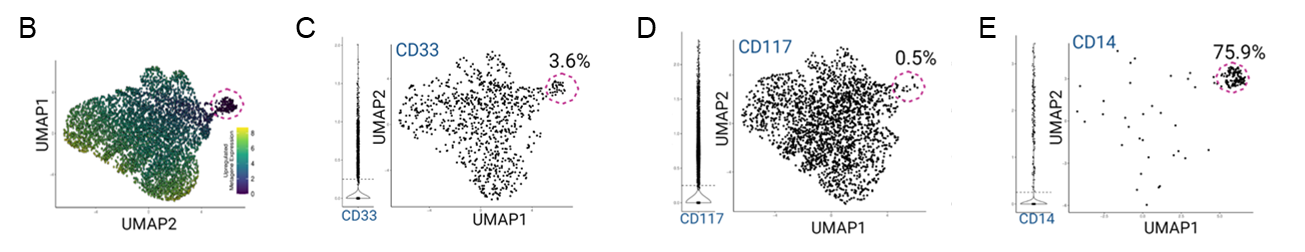


**Supplementary Figure 4**: Single cell RNA sequencing (sc-RNA seq) analysis could differentiate a small population of wild-type cells from the majority of leukemic cells by the -7/del7q genes signature in a single -7 AML sample

**A** Our processing overview of cells and 12 genes selection in sc-RNA seq. **B** 2d plots of single bone marrow cells based on UMAP of the remaining 3 HI genes and 9 upregulated genes. **C, D, E** UMAP of 3 HI genes in CD33, CD117, and CD14 positive cells. The majority of our HI genes signature was enriched in CD33 and CD117-positive leukemia cells but rarely enriched in CD14-positive cells. The cell populations circled by red lines were wild-type cells


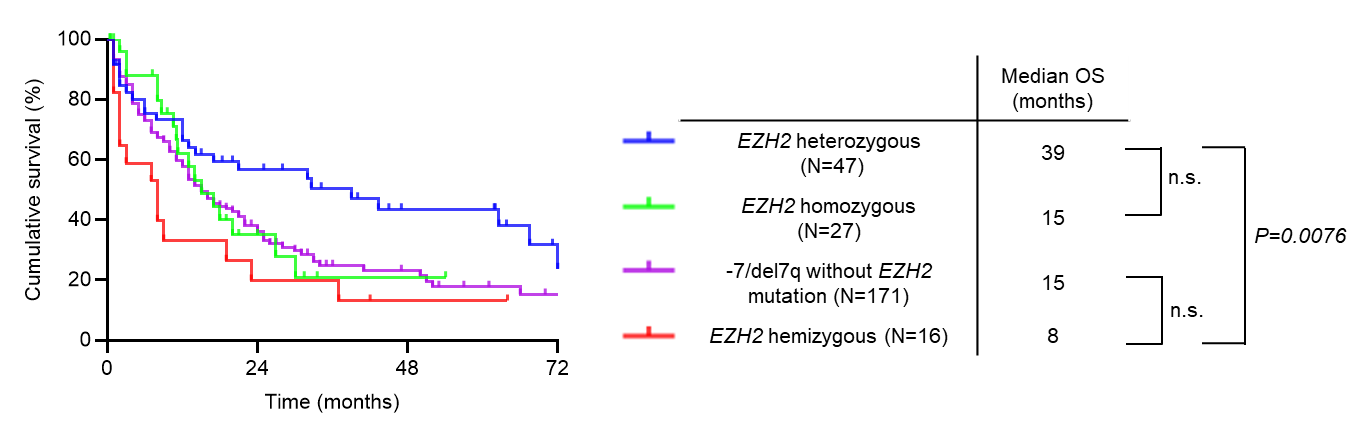


**Supplementary Figure 5:** **Overall survival by *EZH2* mutational zygosity**

The patients with hemizygous EZH2 mutations showed the worst prognosis.

**Table S1. Summary of all the sample sources in our study**

| **Cohorts** | **Total (n=8,142)** |
| --- | --- |
| Cleveland Clinic | 1,667 |
| Munich Leukemia Laboratory (MLL) | 4,573 |
| The Cancer Genome Atlas (TCGA) | 181 |
| German-Austrian Study Group | 1,217 |
| Beat AML Master Trial | 504 |

**Table S2. List of 163 genes on germ line targeted panel**

| Gene Symbol | Cytogenetic band | Gene Category |
| --- | --- | --- |
| *ABL1* | 9q34.12 | Receptors/Kinases/Signaling |
| *ADCY3* | 2p23.3 | Other undefined GLVs |
| *ANGPT2* | 8p23.1 | Other undefined GLVs |
| *APC* | 5q22.2 | Cancer predisposition gene |
| *ASXL1* | 20q11.21 | Histone/Chromatin modification |
| *ATM* | 11q22.3 | DNA repair gene |
| *ATR* | 3q23 | DNA repair gene |
| *ATRX* | Xq21.1 | Histone/Chromatin modification |
| *BAP1* | 3p21.1 | Other undefined GLVs |
| *BARD1* | 2q35 | Cancer predisposition gene |
| *BCAS1* | 20q13.2 | Other undefined GLVs |
| *BCOR* | Xp11.4 | Histone/Chromatin modification |
| *BCORL1* | Xq26.1 | Histone/Chromatin modification |
| *BLM* | 15q26.1 | DNA repair gene |
| *BMPR1A* | 10q23.2 | Cancer predisposition gene |
| *BRCA1* | 17q21.31 | FA gene |
| *BRCA2* | 13q13.1 | FA gene |
| *BRCC3* | Xq28 | Other undefined GLVs |
| *BRIP1* | 17q23.2 | FA gene |
| *BUB1B* | 15q15.1 | Cancer predisposition gene |
| *CALR* | 19p13.13 | Other undefined GLVs |
| *CBL* | 11q23.3 | RASopathies |
| *CBLB* | 3q13.11 | RASopathies |
| *CBLC* | 19q13.32 | RASopathies |
| *CDAN1* | 15q15.2 | Classical IBMFs gene |
| *CDC25B* | 20p13 | Other undefined GLVs |
| *CDH1* | 16q22.1 | Other undefined GLVs |
| *CDK4* | 12q14.1 | Receptors/Kinases/Signaling |
| *CDKN2A* | 9p21.3 | Cancer predisposition gene |
| *CEBPA* | 19q13.11 | MN predisposition gene |
| *CHEK2* | 22q12.1 | Cancer predisposition gene |
| *CSF1R* | 5q32 | Receptors/Kinases/Signaling |
| *CSF2RA* | Xp22.32 and Yp11.3 | Receptors/Kinases/Signaling |
| *CSF2RB* | 22q12.3 | Receptors/Kinases/Signaling |
| *CSF3R* | 1p34.3 | Receptors/Kinases/Signaling |
| *CTC1* | 17p13.1 | Telomerase gene |
| *CUX1* | 7q22.1 | Cancer predisposition gene |
| *DDX41* | 5q35.3 | MN predisposition gene |
| *DKC1* | Xq28 | Telomerase gene |
| *DNAJC21* | 5p13.2 | Classical IBMFs gene |
| *ELANE* | 19p13.3 | Classical IBMFs gene |
| *EP300* | 22q13.2 | Cancer predisposition gene |
| *ERCC6* | 10q11.23 | DNA repair gene |
| *ETNK1* | 12p12.1 | Other undefined GLVs |
| *ETV6* | 12p13.2 | MN predisposition gene |
| *EZH2* | 7q36.1 | Histone/Chromatin modification |
| *FAM175A* | 4q21.23 | Cancer predisposition gene |
| *FANCA* | 16q24.3 | FA gene |
| *FANCB* | Xp22.2 | FA gene |
| *FANCC* | 9q22.32 | FA gene |
| *FANCD2* | 3p25.3 | FA gene |
| *FANCE* | 6p21.31 | FA gene |
| *FANCF* | 11p14.3 | FA gene |
| *FANCG* | 9p13.3 | FA gene |
| *FANCI* | 15q26.1 | FA gene |
| *FANCL* | 2p16.1 | FA gene |
| *FANCM* | 14q21.2 | FA gene |
| *FAT4* | 4q28.1 | Other undefined GLVs |
| *FBXW7* | 4q31.3 | Cancer predisposition gene |
| *FH* | 1q43 | Other undefined GLVs |
| *FLT3* | 13q12.2 | Receptors/Kinases/Signaling |
| *G3BP1* | 5q33.1 | RASopathies |
| *G6PC3* | 17q21.31 | Classical IBMFs gene |
| *GALNT12* | 9q22.33 | Cancer predisposition gene |
| *GAR1* | 4q25 | Telomerase gene |
| *GATA1* | Xp11.23 | Classical IBMFs gene |
| *GATA2* | 3q21.3 | MN predisposition gene |
| *GFI1* | 1p22.1 | Transcription factor |
| *GFI1B* | 9q34.13 | Transcription factor |
| *GIGYF1* | 7q22.1 | Other undefined GLVs |
| *GNA11* | 19p13.3 | Receptors/Kinases/Signaling |
| *GNAS* | 20q13.32 | Receptors/Kinases/Signaling |
| *HAX1* | 1q21.3 | Classical IBMFs gene |
| *HOXB13* | 17q21.32 | Cancer predisposition gene |
| *HRAS* | 11p15.5 | RASopathies |
| *IDH2* | 15q26.1 | DNA methylation |
| *IDH3A* | 15q25.1 | DNA methylation |
| *IKZF1* | 7p12.2 | Transcription factor |
| *JAK2* | 9p24.1 | Receptors/Kinases/Signaling |
| *JAK3* | 19p13.11 | Receptors/Kinases/Signaling |
| *JARID2* | 6p22.3 | Histone/Chromatin modification |
| *KDM6A* | Xp11.3 | Histone/Chromatin modification |
| *KDM6B* | 17p13.1 | Other undefined GLVs |
| *KDR* | 4q12 | Other undefined GLVs |
| *KIT* | 4q12 | Receptors/Kinases/Signaling |
| *KLF1* | 19p13.13 | Classical IBMFs gene |
| *KMT2D* | 12q13.12 | Histone/Chromatin modification |
| *KRAS* | 12p12.1 | RASopathies |
| *LUC7L2* | 7q34 | RNA splicing/ribosome |
| *MFSD11* | 17q25.1 | Other undefined GLVs |
| *MLH1* | 3p22.2 | DNA repair gene |
| *MPL* | 1p34.2 | Receptors/Kinases/Signaling |
| *MRE11A* | 11q21 | DNA repair gene |
| *MSH2* | 2p21-p16.3 | DNA repair gene |
| *MSH6* | 2p16.3 | DNA repair gene |
| *MUTYH* | 1p34.1 | Cancer predisposition gene |
| *NBN* | 8q21.3 | DNA repair gene |
| *NF1* | 17q11.2 | RASopathies |
| *NFE2* | 12q13.13 | Other undefined GLVs |
| *NOTCH1* | 9q34.3 | Receptors/Kinases/Signaling |
| *NRAS* | 1p13.2 | RASopathies |
| *NRP1* | 10p11.22 | Cancer predisposition gene |
| *PALB2* | 16p12.2 | FA gene |
| *PCLO* | 7q21.11 | Other undefined GLVs |
| *PDGFRA* | 4q12 | Other undefined GLVs |
| *PHF6* | Xq26.2 | Cancer predisposition gene |
| *PIGT* | 20q13.12 | GPI-anchor biosynthesis |
| *PLA2G4C* | 19q13.33 | Other undefined GLVs |
| *PMS2* | 7p22.1 | DNA repair gene |
| *POT1* | 7q31.33 | Telomerase gene |
| *PRF1* | 10q22.1 | Classical IBMFs gene |
| *PRPF8* | 17p13.3 | RNA splicing/ribosome |
| *PRSS1* | 7q34 | Other undefined GLVs |
| *PTEN* | 10q23.31 | Receptors/Kinases/Signaling |
| *PTK2* | 8q24.3 | Other undefined GLVs |
| *PTPN11* | 12q24.13 | RASopathies |
| *RAB12* | 18p11.22 | RASopathies |
| *RAD21* | 8q24.11 | Cohesins |
| *RAD50* | 5q31.1 | DNA repair gene |
| *RAD51* | 15q15.1 | DNA repair gene |
| *RAD51C* | 17q22 | FA gene |
| *RAD51D* | 17q12 | DNA repair gene |
| *RAD54L* | 1p34.1 | DNA repair gene |
| *RET* | 10q11.21 | Cancer predisposition gene |
| *RIT1* | 1q22 | RASopathies |
| *RPL11* | 1p36.11 | RNA splicing/ribosome |
| *RPL35A* | 3q29 | RNA splicing/ribosome |
| *RPL5* | 1p22.1 | Classical IBMFs gene |
| *RPS10* | 6p21.31 | Classical IBMFs gene |
| *RPS19* | 19q13.2 | Classical IBMFs gene |
| *RPS24* | 10q22.3 | RNA splicing/ribosome |
| *RTEL1* | 20q13.33 | Telomerase gene |
| *RUNX1* | 21q22.12 | MN predisposition gene |
| *RUNX1T1* | 8q21.3 | Other undefined GLVs |
| *SAMD9* | 7q21.2 | MN predisposition gene |
| *SAMD9L* | 7q21.2 | MN predisposition gene |
| *SBDS* | 7q11.21 | Classical IBMFs gene |
| *SETBP1* | 18q12.3 | Cancer predisposition gene |
| *SETD2* | 3p21.31 | Histone/Chromatin modification |
| *SH2B3* | 12q24.12 | Receptors/Kinases/Signaling |
| *SMAD4* | 18q21.2 | Other undefined GLVs |
| *SMC1A* | Xp11.22 | Cohesins |
| *SRP72* | 4q12 | MN predisposition gene |
| *STAT5B* | 17q21.2 | Receptors/Kinases/Signaling |
| *STK11* | 19p13.3 | Cancer predisposition gene |
| *STK4* | 20q13.12 | Other undefined GLVs |
| *SUZ12* | 17q11.2 | Histone/Chromatin modification |
| *TERT* | 5p15.33 | Telomerase gene |
| *TET2* | 4q24 | DNA methylation |
| *TINF2* | 14q12 | Telomerase gene |
| *TNFAIP3* | 6q23.3 | Other undefined GLVs |
| *TP53* | 17p13.1 | MN predisposition gene |
| *VHL* | 3p25.3 | Cancer predisposition gene |
| *VPS45* | 1q21.2 | Other undefined GLVs |
| *WAS* | Xp11.23 | Other undefined GLVs |
| *WASF2* | 1p36.11 | Other undefined GLVs |
| *WASF3* | 13q12.13 | Other undefined GLVs |
| *WRAP53* | 17p13.1 | Other undefined GLVs |
| *WT1* | 11p13 | Transcription factor |
| *XRCC2* | 7q36.1 | FA gene |
| *XRCC3* | 14q32.33 | DNA repair gene |
| *ZNF276* | 16q24.3 | Other undefined GLVs |
| *ZRSR2* | Xp22.2 | RNA splicing/ribosome |

**Table S3. List of 48 genes on the single cell DNA sequencing panel**

| *ASXL1* | *GNAS* | *NF1* | *SAMD9L* |
| --- | --- | --- | --- |
| *BCOR* | *IDH1* | *NPM1* | *SETBP1* |
| *BRAF* | *IDH2* | *NRAS* | *SF3B1* |
| *CALR* | *JAK2* | *PHF6* | *SMC1A* |
| *CBL* | *KDM6A* | *PIGA* | *SMC3* |
| *CSF3R* | *KIT* | *PPM1D* | *STAG2* |
| *DNMT3A* | *KMT2A* | *PRPF8* | *STAT3* |
| *EP300* | *KRAS* | *PTEN* | *TET2* |
| *ETV6* | *LUC7L2* | *PTPN11* | *TP53* |
| *EZH2* | *MPL* | *RAD21* | *U2AF1* |
| *FLT3* | *MYC* | *RUNX1* | *WT1* |
| *GATA2* | *MYD88* | *SAMD9* | *ZRSR2* |

**Table S5. *EZH2* mutational list in myeloid neoplasms with -7/del7q**

| Diagnosis | Karyotype | Deleted location in chromosome 7 | Mutation | Genotype |
| --- | --- | --- | --- | --- |
| AML | -7 | all | splice site  (c.-7-1G>C) | Hemizygous |
| AML | -7 | all | p.R25X | Hemizygous |
| AML | complex with -7 | all | p.E125V | Hemizygous |
| AML | -7 | all | p.H129R | Hemizygous |
| MDS/MPN-U | del(7q), del(12p) | 7q | p.R168X | Hemizygous |
| CMML | del(7)(q11) | 7q11.1-q11.23 | p.H279fs | Heterozygous |
| MDS | -7, +14 | all | p.E360X | Hemizygous |
| MPN (MF) | -7 | all | p.Q426fs | Hemizygous |
| MDS | complex with der(7)t(6;7)(q13;q21) | 7q21.11-q36.3 | splice site  (c.1411-1G>C) | Hemizygous |
| AML | inv(3)(q21q26),-7 | all | p.Q470X | Hemizygous |
| AML | -7 | all | p.E528V | Hemizygous |
| AML | -7, del(9)(q22q32) | all | splice site  (c.1852-1G>A) | Hemizygous |
| MDS | -7 | all | p.S639L | Hemizygous |
| MDS | -7, +8 | all | p.I659fs | Hemizygous |
| AML | complex with -7 | all | p.G660E | Hemizygous |
| AML | -7, del(9)(q13q34) | all | p.D664N | Hemizygous |
| AML | complex with -7 | all | p.D674X | Hemizygous |
| AML | -7 | all | p.R684H | Hemizygous |
| MDS | -7 | all | p.Y685fs | Hemizygous |
| MDS | -7, +8, der(7)t(1;7)(q12;q11.2) | all | p.G686A | Hemizygous |
| AML | der(7)del(7)(q21q22)del(7)(q31q36),+11 | 7q21-q22,  7q31-q36 | p.R690H | Hemizygous |
| AML | -7 | all | p.R690H | Hemizygous |
| AML | -7 | all | p.R690H | Hemizygous |
| AML | del(7q) | 7q | p.R690H | Hemizygous |
| AML | -7, +13 | all | p.E745SfsX24 | Hemizygous |

**Table S6. *CUX1* mutational list in myeloid neoplasms with -7/del7q**

| Diagnosis | Karyotype | Deleted location in chromosome 7 | Mutation | Genotype |
| --- | --- | --- | --- | --- |
| AML | del(5)(q13q33), -7 | all | p.K546Q | Hemizygous |
| AML | complex with del7q | 7q22-q34 | p.S1134C | Hemizygous |
| AML | complex with -7 | all | p.Q312Rfs*29 | Hemizygous |
| MDS | complex with -7 | all | p.P1106L | Hemizygous |
| CMML | -7 | all | p.S194fs | Hemizygous |
| AML | complex with -7 | all | p.V99I | Hemizygous |
| AML | -7 | all | p.A1381T | Hemizygous |
| AML | -7, del(12)(p11p13) | all | p.E1375X | Hemizygous |
| AML | -7 | all | splice site  (c.3107-3T>C) | Hemizygous |
| MDS | del7q, del20q | q11.21-q31.33 | c.2819del | Hemizygous |
| MDS | complex with -7 | all | p.L157F | Hemizygous |

**Table S7. Germline mutations found in -7/del7q myeloid neoplasm cases**

| Age/Gender | Diagnosis | -7/del7q | | Gene | Variant | ACMG classification |
| --- | --- | --- | --- | --- | --- | --- |
| 65/Male | AML | del7q | *ATM* | | p.Q2729X | Pathogenic |
| 65/Male | CMML/AA | del7q | *BARD1* | | p.Q94X | Pathogenic |
| 68/Female | MDS/AA | -7 | *BLM* | | splice site (c.2074+1G>A) | Pathogenic |
| 23/Male | AML | del7q | *BRCA2* | | p.R2287X | Pathogenic |
| 71/Male | MDS | del7q | *FANCC* | | p.R185X | Pathogenic |
| 50/Female | MDS | -7 | *FANCD2* | | p.Y1267fs | Pathogenic |
| 48/Female | AML | -7 | *FANCE* | | p.R371W | Likely pathogenic |
| 74/Male | AML | -7 | *FANCE* | | p.V471fs | Pathogenic |
| 67/Male | CMML/AA | -7 | *FANCE* | | splice site (c.249-2A) | Pathogenic |
| 83/Female | AML | -7 | *FANCM* | | p.R284X | Pathogenic |
| 50/Female | MDS | del7q | *FANCM* | | p.E860X | Pathogenic |
| 42/Male | AML | -7 | *FANCM* | | p.K659X | Pathogenic |
| 58/Male | AML | del7q | *MRE11A* | | p.L57X | Pathogenic |
| 78/Male | AML | -7 | *RAD50* | | splice site (c.129+2T>A) | Pathogenic |
| 67/Female | MDS | -7 | *RAD51C* | | splice site (c.905-2_905-1delAG) | Pathogenic |
| 83/Male | MDS | -7 | *NF1* | | p.G2518X | Pathogenic |
| 57/Female | AML | -7 | *NF1* | | p.Y2264X | Pathogenic |
|  |  |  | *TP53* | | p.C275Y | Likely pathogenic |
| 82/Male | MDS/MPN-U | -7 | *RIT1* | | p.F46L | Likely pathogenic |
| 58/Male | AML | -7 | *RPS19* | | p.R62Q | Likely pathogenic |
| 17/Male | MDS/AA | -7 | *SBDS* | | p.K62X | Pathogenic |
| 78/Female | MDS | -7 | *SAMD9L* | | p.I327V | Likely pathogenic |
| 77/Male | AML | -7 | *SAMD9L* | | p.L1323fs | Pathogenic |
| 77/Male | AML | -7 | *TP53* | | p.V140M | Likely pathogenic |
| 73/Male | AML | -7 | *CDAN1* | | p.Q107X | Pathogenic |
| 52/Male | AML | -7 | *CSF1R* | | p.Q481X | Pathogenic |
| 68/Male | AML | -7 | *DDX41* | | p.D140_P141delinsGX | Pathogenic |

AA, aplastic anemia; AML, acute myeloid leukemia; CMML, Chronic myelomonocytic leukemia; MDS, myelodysplastic syndrome; MDS/MPN-u, myelodysplastic/myeloproliferative neoplasm, unclassifiable

**Table S8. 304 genes used for haploinsufficient genes analysis of -7/del7q**

| Gene | Location | % of the cases<50^th^%tile | Slope | Coefficient | R squared | P value | FDR |
| --- | --- | --- | --- | --- | --- | --- | --- |
| *AGK* | q34 | 100 | -0.00837 | -0.00837 | 0.310842 | 0.000227 | 0.007423 |
| *ARPC1A* | q22.1 | 100 | -0.0123 | -0.0123 | 0.51052 | 4.69E-07 | 0.000292 |
| *ZNF277* | q31.1 | 100 | -0.0169 | -0.0169 | 0.41155 | 1.08E-05 | 0.001676 |
| *ZNF398* | q36.1 | 100 | -0.0073 | -0.0073 | 0.133665 | 0.015913 | 0.089173 |
| *ABCB8* | q36.1 | 97.91667 | -0.01192 | -0.01192 | 0.161522 | 0.007553 | 0.065247 |
| *CUL1* | q36.1 | 97.91667 | -0.00846 | -0.00846 | 0.289756 | 0.000197 | 0.007125 |
| *GALNT11* | q36.1 | 97.91667 | -0.01293 | -0.01293 | 0.341385 | 3.89E-05 | 0.002423 |
| *GSTK1* | q34 | 97.91667 | -0.01606 | -0.01606 | 0.458361 | 2.22E-06 | 0.000689 |
| *IMPDH1* | q32.1 | 97.91667 | -0.01122 | -0.01122 | 0.180099 | 0.007089 | 0.064847 |
| *KLHDC10* | q32.2 | 97.91667 | -0.00715 | -0.00715 | 0.239249 | 0.001831 | 0.025407 |
| *LMBR1* | q36.3 | 97.91667 | -0.00702 | -0.00702 | 0.111819 | 0.037465 | 0.148428 |
| *MRPS33* | q34 | 97.91667 | -0.00901 | -0.00901 | 0.17756 | 0.00755 | 0.065247 |
| *NDUFB2* | q34 | 97.91667 | -0.00906 | -0.00906 | 0.107393 | 0.041692 | 0.156219 |
| *PDIA4* | q36.1 | 97.91667 | -0.00519 | -0.00519 | 0.053283 | 0.136419 | 0.338241 |
| *POLR2J* | q22.1 | 97.91667 | -0.01313 | -0.01313 | 0.440724 | 5.53E-06 | 0.001146 |
| *POP7* | q22.1 | 97.91667 | -0.01331 | -0.01331 | 0.260177 | 0.00107 | 0.019013 |
| *PSMC2* | q22.1 | 97.91667 | -0.0042 | -0.0042 | 0.080543 | 0.084216 | 0.245111 |
| *SLC37A3* | q34 | 97.91667 | -0.01582 | -0.01582 | 0.244599 | 0.001372 | 0.023069 |
| *SND1* | q32.1 | 97.91667 | -0.0075 | -0.0075 | 0.172975 | 0.008456 | 0.066445 |
| *SSBP1* | q34 | 97.91667 | -0.00832 | -0.00832 | 0.19076 | 0.005437 | 0.052024 |
| *TES* | q31.2 | 97.91667 | -0.01218 | -0.01218 | 0.157378 | 0.012406 | 0.080159 |
| *TRIM56* | q22.1 | 97.91667 | -0.013 | -0.013 | 0.410632 | 1.47E-05 | 0.001823 |
| *ZNHIT1* | q22.1 | 97.91667 | -0.01395 | -0.01395 | 0.403696 | 1.82E-05 | 0.001889 |
| *ZSCAN25* | q22.1 | 97.91667 | -0.01005 | -0.01005 | 0.291518 | 0.000468 | 0.010773 |
| *ALKBH4* | q22.1 | 95.83333 | -0.00728 | -0.00728 | 0.118811 | 0.034069 | 0.140336 |
| *ARF5* | q32.1 | 95.83333 | -0.00623 | -0.00623 | 0.173456 | 0.008356 | 0.066445 |
| *ARPC1B* | q22.1 | 95.83333 | -0.01385 | -0.01385 | 0.168505 | 0.010471 | 0.074865 |
| *CALU* | q32.1 | 95.83333 | -0.00872 | -0.00872 | 0.237352 | 0.001658 | 0.024557 |
| *CHPF2* | q36.1 | 95.83333 | -0.00555 | -0.00555 | 0.035369 | 0.22716 | 0.464781 |
| *COG5* | q22.3 | 95.83333 | -0.0063 | -0.0063 | 0.096626 | 0.061124 | 0.20331 |
| *CPSF4* | q22.1 | 95.83333 | -0.00756 | -0.00756 | 0.148773 | 0.016774 | 0.091522 |
| *DLD* | q31.1 | 95.83333 | -0.00261 | -0.00261 | 0.019135 | 0.401017 | 0.611354 |
| *DMTF1* | q21.12 | 95.83333 | -0.00877 | -0.00877 | 0.254342 | 0.003816 | 0.043569 |
| *ESYT2* | q36.3 | 95.83333 | -0.00706 | -0.00706 | 0.124751 | 0.027411 | 0.123549 |
| *GCC1* | q32.1 | 95.83333 | -0.00894 | -0.00894 | 0.203753 | 0.003923 | 0.043569 |
| *GIMAP2* | q36.1 | 95.83333 | -0.02078 | -0.02078 | 0.3195 | 7.84E-05 | 0.00405 |
| *GNB2* | q22.1 | 95.83333 | -0.01191 | -0.01191 | 0.15649 | 0.013959 | 0.084721 |
| *IRF5* | q32.1 | 95.83333 | -0.00803 | -0.00803 | 0.062879 | 0.123623 | 0.312575 |
| *KDM7A* | q34 | 95.83333 | -0.01301 | -0.01301 | 0.234027 | 0.001808 | 0.025407 |
| *LAMTOR4* | q22.1 | 95.83333 | -0.01193 | -0.01193 | 0.142125 | 0.019641 | 0.100136 |
| *LRWD1* | q22.1 | 95.83333 | -0.00428 | -0.00428 | 0.03105 | 0.289941 | 0.519722 |
| *MDFIC* | q31.1 | 95.83333 | -0.01865 | -0.01865 | 0.276286 | 0.00059 | 0.011463 |
| *MEPCE* | q22.1 | 95.83333 | -0.00536 | -0.00536 | 0.08971 | 0.067721 | 0.216338 |
| *NDUFA5* | q31.32 | 95.83333 | -0.00696 | -0.00696 | 0.108535 | 0.040558 | 0.152891 |
| *NUP205* | q33 | 95.83333 | -0.00796 | -0.00796 | 0.203908 | 0.00443 | 0.047513 |
| *ORC5* | q22.2 | 95.83333 | -0.00905 | -0.00905 | 0.169858 | 0.012501 | 0.080159 |
| *PILRA* | q22.1 | 95.83333 | -0.01879 | -0.01879 | 0.198619 | 0.005045 | 0.049931 |
| *PMPCB* | q22.1 | 95.83333 | -0.00954 | -0.00954 | 0.253869 | 0.00126 | 0.021762 |
| *PMS2P1* | q22.1 | 95.83333 | -0.00846 | -0.00846 | 0.313739 | 0.000255 | 0.007652 |
| *PPP1R35* | q22.1 | 95.83333 | -0.01099 | -0.01099 | 0.29613 | 0.000413 | 0.010017 |
| *TMEM209* | q32.2 | 95.83333 | -0.00796 | -0.00796 | 0.145349 | 0.018195 | 0.095104 |
| *TMEM243* | q21.12 | 95.83333 | -0.02582 | -0.02582 | 0.45703 | 2.99E-05 | 0.002326 |
| *TNPO3* | q32.1 | 95.83333 | -0.00318 | -0.00318 | 0.035188 | 0.252812 | 0.493316 |
| *TRIM4* | q22.1 | 95.83333 | -0.01672 | -0.01672 | 0.380898 | 3.67E-05 | 0.002423 |
| *TSC22D4* | q22.1 | 95.83333 | -0.01094 | -0.01094 | 0.182292 | 0.00751 | 0.065247 |
| *WDR91* | q33 | 95.83333 | -0.00419 | -0.00419 | 0.048367 | 0.184598 | 0.40317 |
| *ZNF3* | q22.1 | 95.83333 | -0.00667 | -0.00667 | 0.120219 | 0.032957 | 0.137577 |
| *ZNF394* | q22.1 | 95.83333 | -0.01394 | -0.01394 | 0.288132 | 0.000512 | 0.011376 |
| *ZNF655* | q22.1 | 95.83333 | -0.01181 | -0.01181 | 0.2835 | 0.00058 | 0.011463 |
| *ZNF800* | q31.33 | 95.83333 | -0.0036 | -0.0036 | 0.033024 | 0.261754 | 0.502943 |
| *ABCF2* | q36.1 | 93.75 | -0.00888 | -0.00888 | 0.135069 | 0.01533 | 0.088103 |
| *ADCK2* | q34 | 93.75 | -0.003 | -0.003 | 0.015801 | 0.452054 | 0.65403 |
| *AKAP9* | q21.2 | 93.75 | -0.00969 | -0.00969 | 0.305112 | 0.001045 | 0.019013 |
| *ANKIB1* | q21.2 | 93.75 | -0.01014 | -0.01014 | 0.455402 | 2.27E-05 | 0.00202 |
| *AP4M1* | q22.1 | 93.75 | -0.00564 | -0.00564 | 0.055217 | 0.155574 | 0.356311 |
| *ATP6V1F* | q32.1 | 93.75 | -0.00828 | -0.00828 | 0.171274 | 0.008819 | 0.066445 |
| *ATXN7L1* | q22.3 | 93.75 | -0.00632 | -0.00632 | 0.112028 | 0.04288 | 0.158759 |
| *BET1* | q21.3 | 93.75 | -0.01136 | -0.01136 | 0.209032 | 0.005057 | 0.049931 |
| *CAPZA2* | q31.2 | 93.75 | -0.00441 | -0.00441 | 0.087013 | 0.0683 | 0.216338 |
| *CASD1* | q21.3 | 93.75 | -0.01162 | -0.01162 | 0.298814 | 0.000562 | 0.011463 |
| *CDK5* | q36.1 | 93.75 | -0.00339 | -0.00339 | 0.023831 | 0.322951 | 0.548841 |
| *COPS6* | q22.1 | 93.75 | -0.00721 | -0.00721 | 0.229257 | 0.002358 | 0.03055 |
| *CUX1* | q22.1 | 93.75 | -0.00495 | -0.00495 | 0.111499 | 0.040476 | 0.152891 |
| *EPHB6* | q34 | 93.75 | -0.00711 | -0.00711 | 0.087284 | 0.067852 | 0.216338 |
| *EXOC4* | q33 | 93.75 | -0.00424 | -0.00424 | 0.04877 | 0.182734 | 0.401628 |
| *GIGYF1* | q22.1 | 93.75 | -0.01045 | -0.01045 | 0.299343 | 0.000379 | 0.009811 |
| *HIPK2* | q34 | 93.75 | -0.00745 | -0.00745 | 0.079675 | 0.081683 | 0.241342 |
| *ING3* | q31.31 | 93.75 | -0.00661 | -0.00661 | 0.079015 | 0.083012 | 0.243554 |
| *LMTK2* | q21.3 | 93.75 | -0.01166 | -0.01166 | 0.17074 | 0.012252 | 0.080159 |
| *METTL2B* | q32.1 | 93.75 | -0.00433 | -0.00433 | 0.063014 | 0.123208 | 0.312575 |
| *NRF1* | q32.2 | 93.75 | -0.00329 | -0.00329 | 0.055487 | 0.154535 | 0.356311 |
| *NUB1* | q36.1 | 93.75 | -0.00866 | -0.00866 | 0.134803 | 0.015439 | 0.088103 |
| *PARP12* | q34 | 93.75 | -0.01095 | -0.01095 | 0.1436 | 0.017359 | 0.093892 |
| *PDAP1* | q22.1 | 93.75 | -0.00821 | -0.00821 | 0.156472 | 0.013965 | 0.084721 |
| *RBM28* | q32.1 | 93.75 | -0.00556 | -0.00556 | 0.137564 | 0.0201 | 0.100611 |
| *RHEB* | q36.1 | 93.75 | -0.00132 | -0.00132 | 0.003842 | 0.692938 | 0.816302 |
| *SLC25A13* | q21.3 | 93.75 | -0.00595 | -0.00595 | 0.096349 | 0.065404 | 0.214112 |
| *ST7* | q31.2 | 93.75 | -0.00443 | -0.00443 | 0.042161 | 0.209846 | 0.435081 |
| *SYPL1* | q22.3 | 93.75 | -0.00139 | -0.00139 | 0.005515 | 0.662253 | 0.801544 |
| *WASL* | q31.32 | 93.75 | -0.00295 | -0.00295 | 0.032094 | 0.275172 | 0.509009 |
| *WDR60* | q36.3 | 93.75 | -0.01186 | -0.01186 | 0.200007 | 0.004876 | 0.049931 |
| *ACTR3B* | q36.1 | 91.66667 | -0.00985 | -0.00985 | 0.179672 | 0.004618 | 0.048684 |
| *ATP6V0E2* | q36.1 | 91.66667 | -0.0181 | -0.0181 | 0.307872 | 0.000113 | 0.0044 |
| *BRAF* | q34 | 91.66667 | -0.0085 | -0.0085 | 0.124232 | 0.027757 | 0.124209 |
| *BUD31* | q22.1 | 91.66667 | -0.00981 | -0.00981 | 0.155846 | 0.014175 | 0.084721 |
| *C7orf43* | q22.1 | 91.66667 | -0.01419 | -0.01419 | 0.209034 | 0.003904 | 0.043569 |
| *CNOT4* | q33 | 91.66667 | -0.00565 | -0.00565 | 0.07073 | 0.106552 | 0.283178 |
| *COPG2* | q32.2 | 91.66667 | -0.00045 | -0.00045 | 0.000235 | 0.927157 | 0.943849 |
| *EZH2* | q36.1 | 91.66667 | 0.000308 | 0.000308 | 0.00025 | 0.919811 | 0.942628 |
| *FAM185A* | q22.1 | 91.66667 | -0.0031 | -0.0031 | 0.034327 | 0.265435 | 0.50474 |
| *FASTK* | q36.1 | 91.66667 | -0.01132 | -0.01132 | 0.116228 | 0.025274 | 0.117316 |
| *HBP1* | q22.3 | 91.66667 | -0.00752 | -0.00752 | 0.069226 | 0.115636 | 0.29969 |
| *KRIT1* | q21.2 | 91.66667 | -0.0044 | -0.0044 | 0.114373 | 0.058333 | 0.195069 |
| *LRCH4* | q22.1 | 91.66667 | -0.01008 | -0.01008 | 0.163553 | 0.011791 | 0.08011 |
| *PRRT4* | q32.1 | 91.66667 | -0.00323 | -0.00323 | 0.004408 | 0.688002 | 0.815037 |
| *RINT1* | q22.3 | 91.66667 | -0.00494 | -0.00494 | 0.0585 | 0.149233 | 0.356311 |
| *SRPK2* | q22.3 | 91.66667 | -0.01306 | -0.01306 | 0.244564 | 0.001863 | 0.025407 |
| *TBXAS1* | q34 | 91.66667 | -0.01048 | -0.01048 | 0.071884 | 0.09889 | 0.272167 |
| *TECPR1* | q21.3 | 91.66667 | -0.01307 | -0.01307 | 0.193369 | 0.007284 | 0.065247 |
| *TMEM168* | q31.1 | 91.66667 | -0.00771 | -0.00771 | 0.115357 | 0.034397 | 0.140755 |
| *UBE3C* | q36.3 | 91.66667 | -0.00529 | -0.00529 | 0.055401 | 0.149176 | 0.356311 |
| *ZC3HC1* | q32.2 | 91.66667 | -0.0084 | -0.0084 | 0.176116 | 0.008719 | 0.066445 |
| *ZKSCAN1* | q22.1 | 91.66667 | -0.00888 | -0.00888 | 0.347291 | 9.91E-05 | 0.0044 |
| *ZKSCAN5* | q22.1 | 91.66667 | -0.00541 | -0.00541 | 0.13418 | 0.023704 | 0.113058 |
| *ZNF282* | q36.1 | 91.66667 | -0.00461 | -0.00461 | 0.034617 | 0.232254 | 0.470561 |
| *AP1S1* | q22.1 | 89.58333 | -0.00299 | -0.00299 | 0.019179 | 0.406997 | 0.614447 |
| *BCAP29* | q22.3 | 89.58333 | -0.00181 | -0.00181 | 0.010566 | 0.544911 | 0.73204 |
| *BRI3* | q21.3 | 89.58333 | -0.00703 | -0.00703 | 0.058348 | 0.155814 | 0.356311 |
| *CASP2* | q34 | 89.58333 | 0.002206 | 0.002206 | 0.021505 | 0.373012 | 0.589389 |
| *CCDC71L* | q22.3 | 89.58333 | -0.00601 | -0.00601 | 0.052987 | 0.170494 | 0.379216 |
| *CDK14* | q21.13 | 89.58333 | -0.00874 | -0.00874 | 0.117961 | 0.054283 | 0.187577 |
| *CEP41* | q32.2 | 89.58333 | -0.00571 | -0.00571 | 0.033138 | 0.274022 | 0.509009 |
| *CHCHD3* | q33 | 89.58333 | -0.00459 | -0.00459 | 0.025549 | 0.337763 | 0.561173 |
| *DUS4L* | q22.3 | 89.58333 | -0.00895 | -0.00895 | 0.182065 | 0.008448 | 0.066445 |
| *KMT2C* | q36.1 | 89.58333 | -0.00383 | -0.00383 | 0.03045 | 0.263069 | 0.502943 |
| *LINC01003* | q36.1 | 89.58333 | -0.00355 | -0.00355 | 0.061462 | 0.108953 | 0.284743 |
| *LRRC4* | q32.1 | 89.58333 | -0.00545 | -0.00545 | 0.015107 | 0.456007 | 0.655068 |
| *LUC7L2* | q34 | 89.58333 | -0.00349 | -0.00349 | 0.021476 | 0.373343 | 0.589389 |
| *MKLN1* | q32.3 | 89.58333 | -0.00766 | -0.00766 | 0.165926 | 0.012339 | 0.080159 |
| *NOM1* | q36.3 | 89.58333 | -0.0062 | -0.0062 | 0.074206 | 0.0934 | 0.262872 |
| *PAXIP1* | q36.2 | 89.58333 | -0.00311 | -0.00311 | 0.019659 | 0.381929 | 0.589788 |
| *POLR2J3* | q22.1 | 89.58333 | -0.0133 | -0.0133 | 0.167872 | 0.010632 | 0.075147 |
| *PRKAG2* | q36.1 | 89.58333 | -0.0087 | -0.0087 | 0.050855 | 0.145946 | 0.356311 |
| *PRKRIP1* | q22.1 | 89.58333 | -0.00111 | -0.00111 | 0.040649 | 0.22481 | 0.463019 |
| *REPIN1* | q36.1 | 89.58333 | -0.00101 | -0.00101 | 0.001661 | 0.795241 | 0.87364 |
| *SLC4A2* | q36.1 | 89.58333 | -0.00297 | -0.00297 | 0.013769 | 0.453636 | 0.654667 |
| *SMARCD3* | q36.1 | 89.58333 | -0.00406 | -0.00406 | 0.013625 | 0.45602 | 0.655068 |
| *STAG3L5P* | q22.1 | 89.58333 | -0.009 | -0.009 | 0.123037 | 0.030837 | 0.132281 |
| *TPI1P2* | q32.1 | 89.58333 | -0.00128 | -0.00128 | 0.021372 | 0.374517 | 0.589745 |
| *ZBED6CL* | q36.1 | 89.58333 | -0.00236 | -0.00236 | 0.015813 | 0.421695 | 0.627499 |
| *AKR1B1* | q33 | 87.5 | -0.01031 | -0.01031 | 0.15676 | 0.013869 | 0.084721 |
| *ARMC10* | q22.1 | 87.5 | -0.00209 | -0.00209 | 0.005937 | 0.645663 | 0.799163 |
| *CNPY4* | q22.1 | 87.5 | -0.01031 | -0.01031 | 0.150329 | 0.016165 | 0.08942 |
| *EPHA1* | q35 | 87.5 | -0.00706 | -0.00706 | 0.184827 | 0.005026 | 0.049931 |
| *GATAD1* | q21.2 | 87.5 | -0.0037 | -0.0037 | 0.05402 | 0.200517 | 0.424223 |
| *GIMAP4* | q36.1 | 87.5 | -0.02532 | -0.02532 | 0.318618 | 8.06E-05 | 0.00405 |
| *LRRN3* | q31.1 | 87.5 | -0.00746 | -0.00746 | 0.151307 | 0.014389 | 0.084721 |
| *MCM7* | q22.1 | 87.5 | -0.00142 | -0.00142 | 0.002893 | 0.748411 | 0.847447 |
| *MGAM* | q34 | 87.5 | -0.00365 | -0.00365 | 0.006156 | 0.634951 | 0.793051 |
| *ORAI2* | q22.1 | 87.5 | -0.00809 | -0.00809 | 0.053918 | 0.160664 | 0.366055 |
| *PIK3CG* | q22.3 | 87.5 | -0.00324 | -0.00324 | 0.010743 | 0.541544 | 0.73044 |
| *POT1* | q31.33 | 87.5 | -0.00383 | -0.00383 | 0.031422 | 0.273848 | 0.509009 |
| *RAB19* | q34 | 87.5 | -0.00254 | -0.00254 | 0.055606 | 0.148404 | 0.356311 |
| *SAP25* | q22.1 | 87.5 | -0.00766 | -0.00766 | 0.08597 | 0.074008 | 0.226539 |
| *SLC12A9* | q22.1 | 87.5 | -0.01646 | -0.01646 | 0.185881 | 0.006884 | 0.063907 |
| *SLC25A40* | q21.12 | 87.5 | -0.00216 | -0.00216 | 0.005654 | 0.687668 | 0.815037 |
| *TP53TG1* | q21.12 | 87.5 | -0.00684 | -0.00684 | 0.217707 | 0.008146 | 0.066445 |
| *TPK1* | q35 | 87.5 | -0.0109 | -0.0109 | 0.103537 | 0.040214 | 0.152891 |
| *ZNF862* | q36.1 | 87.5 | -0.01099 | -0.01099 | 0.104171 | 0.034778 | 0.140888 |
| *DNAJC2* | q22.1 | 85.41667 | -0.00241 | -0.00241 | 0.009155 | 0.567703 | 0.7523 |
| *GIMAP1* | q36.1 | 85.41667 | -0.01598 | -0.01598 | 0.264501 | 0.000419 | 0.010017 |
| *GNG11* | q21.3 | 85.41667 | -0.02625 | -0.02625 | 0.326778 | 0.000271 | 0.007652 |
| *GPC2* | q22.1 | 85.41667 | -0.00041 | -0.00041 | 0.006899 | 0.620047 | 0.790305 |
| *IFRD1* | q31.1 | 85.41667 | -0.0007 | -0.0007 | 0.001262 | 0.830021 | 0.890126 |
| *MOSPD3* | q22.1 | 85.41667 | -0.00314 | -0.00314 | 0.028554 | 0.310498 | 0.539137 |
| *NOS3* | q36.1 | 85.41667 | -0.0012 | -0.0012 | 0.017784 | 0.393921 | 0.602011 |
| *PEX1* | q21.2 | 85.41667 | -0.01207 | -0.01207 | 0.407447 | 8.47E-05 | 0.00405 |
| *PUS7* | q22.3 | 85.41667 | -0.00585 | -0.00585 | 0.044559 | 0.209794 | 0.435081 |
| *RBM48* | q21.2 | 85.41667 | -0.00832 | -0.00832 | 0.153203 | 0.02674 | 0.122183 |
| *SAMD9* | q21.2 | 85.41667 | -0.01437 | -0.01437 | 0.19313 | 0.011849 | 0.08011 |
| *SMURF1* | q22.1 | 85.41667 | -0.0083 | -0.0083 | 0.05161 | 0.170172 | 0.379216 |
| *TAF6* | q22.1 | 85.41667 | -0.0082 | -0.0082 | 0.131563 | 0.025217 | 0.117316 |
| *TFEC* | q31.2 | 85.41667 | -0.00329 | -0.00329 | 0.00799 | 0.588411 | 0.765673 |
| *THAP5* | q31.1 | 85.41667 | -0.0086 | -0.0086 | 0.118685 | 0.03174 | 0.1343 |
| *TMEM140* | q33 | 85.41667 | -0.01026 | -0.01026 | 0.11781 | 0.034882 | 0.140888 |
| *TMUB1* | q36.1 | 85.41667 | -0.00978 | -0.00978 | 0.152273 | 0.009684 | 0.070865 |
| *TRIM24* | q33 | 85.41667 | -0.00371 | -0.00371 | 0.028423 | 0.311628 | 0.539137 |
| *XRCC2* | q36.1 | 85.41667 | -0.00553 | -0.00553 | 0.024925 | 0.311961 | 0.539137 |
| *ZNF789* | q22.1 | 85.41667 | -0.00545 | -0.00545 | 0.078849 | 0.087691 | 0.247928 |
| *FIS1* | q22.1 | 83.33333 | -0.01651 | -0.01651 | 0.227357 | 0.002473 | 0.031391 |
| *HILPDA* | q32.1 | 83.33333 | -0.00558 | -0.00558 | 0.02822 | 0.306663 | 0.538826 |
| *IMMP2L* | q31.1 | 83.33333 | -0.00231 | -0.00231 | 0.01282 | 0.492529 | 0.692687 |
| *SRRT* | q22.1 | 83.33333 | -0.00104 | -0.00104 | 0.008361 | 0.58508 | 0.764538 |
| *TRRAP* | q22.1 | 83.33333 | -0.00465 | -0.00465 | 0.073192 | 0.100422 | 0.273958 |
| *UBE2H* | q32.2 | 83.33333 | -0.0134 | -0.0134 | 0.13779 | 0.021764 | 0.106594 |
| *CBLL1* | q22.3 | 81.25 | -0.00255 | -0.00255 | 0.011409 | 0.529201 | 0.723435 |
| *DNAJB6* | q36.3 | 81.25 | -0.00075 | -0.00075 | 0.001561 | 0.811264 | 0.878115 |
| *FAM3C* | q31.31 | 81.25 | -0.00781 | -0.00781 | 0.061941 | 0.126554 | 0.316132 |
| *GTPBP10* | q21.13 | 81.25 | -0.00812 | -0.00812 | 0.153212 | 0.026735 | 0.122183 |
| *LINC01004* | q22.3 | 81.25 | -0.00462 | -0.00462 | 0.032252 | 0.287521 | 0.519525 |
| *NAMPT* | q22.3 | 81.25 | -0.00491 | -0.00491 | 0.016593 | 0.44736 | 0.651657 |
| *NCAPG2* | q36.3 | 81.25 | 0.001191 | 0.001191 | 0.001482 | 0.815998 | 0.878115 |
| *PILRB* | q22.1 | 81.25 | -0.00456 | -0.00456 | 0.06363 | 0.126549 | 0.316132 |
| *PLOD3* | q22.1 | 81.25 | -0.00167 | -0.00167 | 0.006836 | 0.621662 | 0.790744 |
| *PNPLA8* | q31.1 | 81.25 | 0.000442 | 0.000442 | 0.000371 | 0.907325 | 0.939028 |
| *RBM33* | q36.3 | 81.25 | -0.00267 | -0.00267 | 0.010911 | 0.526834 | 0.721818 |
| *ZNF746* | q36.1 | 81.25 | -0.00517 | -0.00517 | 0.048581 | 0.155529 | 0.356311 |
| *ZSCAN21* | q22.1 | 81.25 | -0.00383 | -0.00383 | 0.023236 | 0.36091 | 0.585273 |
| *ZYX* | q34 | 81.25 | -0.00266 | -0.00266 | 0.008413 | 0.57867 | 0.759351 |
| *CPED1* | q31.31 | 79.16667 | -0.00693 | -0.00693 | 0.065284 | 0.116436 | 0.300511 |
| *CROT* | q21.12 | 79.16667 | -0.00606 | -0.00606 | 0.087576 | 0.106005 | 0.282984 |
| *DBF4* | q21.12 | 79.16667 | -0.00268 | -0.00268 | 0.013373 | 0.535588 | 0.728131 |
| *KRBA1* | q36.1 | 79.16667 | -0.00741 | -0.00741 | 0.091917 | 0.048123 | 0.173174 |
| *SLC35B4* | q33 | 79.16667 | -0.0088 | -0.0088 | 0.132096 | 0.024901 | 0.117316 |
| *SRI* | q21.12 | 79.16667 | 0.0006 | 0.0006 | 0.00097 | 0.8679 | 0.91359 |
| *ZNF212* | q36.1 | 79.16667 | -0.00213 | -0.00213 | 0.006769 | 0.59993 | 0.775793 |
| *BPGM* | q33 | 77.08333 | -0.02043 | -0.02043 | 0.122709 | 0.031077 | 0.132395 |
| *GIMAP6* | q36.1 | 77.08333 | -0.02173 | -0.02173 | 0.288196 | 0.000206 | 0.007125 |
| *GIMAP8* | q36.1 | 77.08333 | -0.01176 | -0.01176 | 0.149927 | 0.010312 | 0.074585 |
| *HEPACAM2* | q21.3 | 77.08333 | -0.01718 | -0.01718 | 0.123004 | 0.035977 | 0.143448 |
| *KMT2E* | q22.3 | 77.08333 | -0.00392 | -0.00392 | 0.019276 | 0.412495 | 0.619177 |
| *LSMEM1* | q31.1 | 77.08333 | -0.00054 | -0.00054 | 0.002093 | 0.782132 | 0.86603 |
| *PDK4* | q21.3 | 77.08333 | -0.00242 | -0.00242 | 0.001985 | 0.796389 | 0.87364 |
| *SAMD9L* | q21.2 | 77.08333 | -0.00589 | -0.00589 | 0.035637 | 0.300792 | 0.532325 |
| *UBN2* | q34 | 77.08333 | 9.03E-05 | 9.03E-05 | 2.53E-05 | 0.975752 | 0.982067 |
| *ZCWPW1* | q22.1 | 77.08333 | -0.00168 | -0.00168 | 0.011747 | 0.517162 | 0.713247 |
| *AHCYL2* | q32.1 | 75 | -0.00756 | -0.00756 | 0.069123 | 0.105865 | 0.282984 |
| *DNAJB9* | q31.1 | 75 | -0.00132 | -0.00132 | 0.003048 | 0.738507 | 0.846947 |
| *DPY19L2P2* | q22.1 | 75 | 0.003554 | 0.003554 | 0.013204 | 0.492112 | 0.692687 |
| *FAM133B* | q21.2 | 75 | -0.0011 | -0.0011 | 0.039324 | 0.2766 | 0.509009 |
| *PRKAR2B* | q22.3 | 75 | -0.00773 | -0.00773 | 0.061775 | 0.137983 | 0.340578 |
| *RASA4* | q22.1 | 75 | -0.0028 | -0.0028 | 0.082823 | 0.079761 | 0.238517 |
| *TMEM176A* | q36.1 | 75 | -0.00035 | -0.00035 | 0.000117 | 0.945078 | 0.958954 |
| *ZC3HAV1* | q34 | 75 | -0.00416 | -0.00416 | 0.033656 | 0.2636 | 0.502943 |
| *CLEC5A* | q34 | 72.91667 | 0.00336 | 0.00336 | 0.004884 | 0.672469 | 0.808845 |
| *CREB3L2* | q33 | 72.91667 | 0.004451 | 0.004451 | 0.045983 | 0.196076 | 0.417669 |
| *GIMAP7* | q36.1 | 72.91667 | -0.02163 | -0.02163 | 0.211458 | 0.00192 | 0.025407 |
| *KCNH2* | q36.1 | 72.91667 | -0.00832 | -0.00832 | 0.02006 | 0.364955 | 0.588087 |
| *MKRN1* | q34 | 72.91667 | -0.01098 | -0.01098 | 0.080274 | 0.080494 | 0.239557 |
| *TTC26* | q34 | 72.91667 | 0.001984 | 0.001984 | 0.0059 | 0.642098 | 0.798069 |
| *ZNF467* | q36.1 | 72.91667 | -0.00303 | -0.00303 | 0.010769 | 0.50782 | 0.703484 |
| *ZNF786* | q36.1 | 72.91667 | -0.0042 | -0.0042 | 0.070914 | 0.084331 | 0.245111 |
| *ACTR3C* | q36.1 | 70.83333 | -0.00021 | -0.00021 | 0.000513 | 0.88538 | 0.924004 |
| *DOCK4* | q31.1 | 70.83333 | -0.00597 | -0.00597 | 0.015885 | 0.444546 | 0.649079 |
| *LRRC61* | q36.1 | 70.83333 | -3.1E-05 | -3.1E-05 | 8.15E-06 | 0.985508 | 0.987095 |
| *PVRIG* | q22.1 | 70.83333 | -0.01866 | -0.01866 | 0.342657 | 0.000113 | 0.0044 |
| *SERPINE1* | q22.1 | 70.83333 | -0.00248 | -0.00248 | 0.004736 | 0.681395 | 0.813489 |
| *STAG3* | q22.1 | 70.83333 | -0.00139 | -0.00139 | 0.002868 | 0.749467 | 0.847447 |
| *TMEM176B* | q36.1 | 70.83333 | -0.0056 | -0.0056 | 0.014349 | 0.444206 | 0.649079 |
| *ABCB4* | q21.12 | 68.75 | -0.0088 | -0.0088 | 0.138271 | 0.039417 | 0.152328 |
| *AGAP3* | q36.1 | 68.75 | -0.00888 | -0.00888 | 0.099431 | 0.039429 | 0.152328 |
| *ASNS* | q21.3 | 68.75 | -0.00223 | -0.00223 | 0.004963 | 0.683067 | 0.813923 |
| *PEG10* | q21.3 | 68.75 | -0.00012 | -0.00012 | 4.07E-05 | 0.970545 | 0.979998 |
| *RELN* | q22.1 | 68.75 | -0.02408 | -0.02408 | 0.167327 | 0.010772 | 0.075281 |
| *CALD1* | q33 | 66.66667 | -0.00661 | -0.00661 | 0.017404 | 0.4298 | 0.635001 |
| *KIAA1147* | q34 | 66.66667 | -0.01354 | -0.01354 | 0.132814 | 0.022552 | 0.108741 |
| *KIAA1324L* | q21.12 | 66.66667 | -0.01722 | -0.01722 | 0.193269 | 0.013338 | 0.083803 |
| *KIAA1549* | q34 | 66.66667 | -0.00326 | -0.00326 | 0.042345 | 0.208832 | 0.435081 |
| *PON2* | q21.3 | 66.66667 | 0.002138 | 0.002138 | 0.007561 | 0.61407 | 0.785909 |
| *STEAP4* | q21.12 | 66.66667 | -0.00819 | -0.00819 | 0.025626 | 0.389658 | 0.596964 |
| *ZNF777* | q36.1 | 66.66667 | -0.00559 | -0.00559 | 0.028193 | 0.281806 | 0.515538 |
| *ACHE* | q22.1 | 64.58333 | -0.00944 | -0.00944 | 0.031817 | 0.283965 | 0.516452 |
| *AOC1* | q36.1 | 64.58333 | -0.00362 | -0.00362 | 0.010698 | 0.509229 | 0.703868 |
| *INSIG1* | q36.2 | 64.58333 | -0.00144 | -0.00144 | 0.002459 | 0.75816 | 0.854303 |
| *KEL* | q34 | 64.58333 | -0.01336 | -0.01336 | 0.047965 | 0.180393 | 0.397888 |
| *MEST* | q32.2 | 64.58333 | -0.00248 | -0.00248 | 0.002997 | 0.744095 | 0.847447 |
| *MET* | q31.2 | 64.58333 | -0.00956 | -0.00956 | 0.054039 | 0.154421 | 0.356311 |
| *NAPEPLD* | q22.1 | 64.58333 | -0.00279 | -0.00279 | 0.007505 | 0.605042 | 0.776572 |
| *PLXNA4* | q32.3 | 64.58333 | -0.01474 | -0.01474 | 0.224919 | 0.003023 | 0.036863 |
| *SH2B2* | q22.1 | 64.58333 | -0.01087 | -0.01087 | 0.179379 | 0.008058 | 0.066445 |
| *TSPAN33* | q32.1 | 64.58333 | -0.00402 | -0.00402 | 0.014357 | 0.467492 | 0.666927 |
| *CDK6* | q21.2 | 62.5 | 0.00296 | 0.00296 | 0.019675 | 0.443854 | 0.649079 |
| *MIR3609* | q22.1 | 62.5 | -0.00459 | -0.00459 | 0.028217 | 0.313414 | 0.539137 |
| *TFR2* | q22.1 | 62.5 | -0.00806 | -0.00806 | 0.026716 | 0.326826 | 0.551482 |
| *TRIP6* | q22.1 | 62.5 | -0.00077 | -0.00077 | 0.000777 | 0.868058 | 0.91359 |
| *PTPRN2* | q36.3 | 60.41667 | -0.00109 | -0.00109 | 0.001204 | 0.83392 | 0.892768 |
| *ZNF783* | q36.1 | 60.41667 | -0.00604 | -0.00604 | 0.050482 | 0.147474 | 0.356311 |
| *AGFG2* | q22.1 | 58.33333 | -0.00339 | -0.00339 | 0.00505 | 0.67159 | 0.808845 |
| *ARHGEF5* | q35 | 58.33333 | -0.00341 | -0.00341 | 0.01785 | 0.404979 | 0.613826 |
| *SSPO* | q36.1 | 58.33333 | -0.00011 | -0.00011 | 2.65E-05 | 0.973865 | 0.981757 |
| *CLDN12* | q21.13 | 56.25 | 0.003889 | 0.003889 | 0.025556 | 0.38213 | 0.589788 |
| *CYP51A1* | q21.2 | 56.25 | 0.005611 | 0.005611 | 0.067844 | 0.149922 | 0.356311 |
| *EPHB4* | q22.1 | 56.25 | -0.00094 | -0.00094 | 0.000722 | 0.87278 | 0.916028 |
| *FBXL13* | q22.1 | 56.25 | -0.00104 | -0.00104 | 0.003886 | 0.710043 | 0.828605 |
| *CLDN15* | q22.1 | 54.16667 | -0.00139 | -0.00139 | 0.002153 | 0.782046 | 0.86603 |
| *FAM71F2* | q32.1 | 54.16667 | -0.00083 | -0.00083 | 0.002088 | 0.782375 | 0.86603 |
| *SEMA3C* | q21.11 | 54.16667 | -0.00538 | -0.00538 | 0.032724 | 0.32178 | 0.548348 |
| *STRIP2* | q32.1 | 54.16667 | -0.00071 | -0.00071 | 0.000257 | 0.922882 | 0.942628 |
| *ADAM22* | q21.12 | 47.91667 | -0.00307 | -0.00307 | 0.014485 | 0.518976 | 0.714166 |
| *CD36* | q21.11 | 47.91667 | 0.006181 | 0.006181 | 0.02111 | 0.427529 | 0.63315 |
| *COL1A2* | q21.3 | 47.91667 | 0.003606 | 0.003606 | 0.004231 | 0.706231 | 0.826292 |
| *PPP1R9A* | q21.3 | 45.83333 | 0.005184 | 0.005184 | 0.011358 | 0.536148 | 0.728131 |
| *FZD1* | q21.13 | 33.33333 | 0.012447 | 0.012447 | 0.150163 | 0.028431 | 0.126315 |
| *HGF* | q21.11 | 33.33333 | 0.016507 | 0.016507 | 0.106668 | 0.068088 | 0.216338 |
| *AASS* | q31.32 | 29.16667 | -0.00291 | -0.00291 | 0.004749 | 0.67679 | 0.811105 |
| *ABCB1* | q21.12 | 20.83333 | -0.00874 | -0.00874 | 0.05369 | 0.209753 | 0.435081 |
| *GNAI1* | q21.11 | 14.58333 | 0.017083 | 0.017083 | 0.119189 | 0.052962 | 0.185071 |
| *ARHGEF34P* | q35 | 0 | 0.000622 | 0.000622 | 0.004899 | 0.663658 | 0.801544 |
| *CADPS2* | q31.32 | 0 | 0.001229 | 0.001229 | 0.002761 | 0.750712 | 0.847447 |
| *CAV1* | q31.2 | 0 | 0.001065 | 0.001065 | 0.004448 | 0.686672 | 0.815037 |
| *CAV2* | q31.2 | 0 | 0.001628 | 0.001628 | 0.003027 | 0.739376 | 0.846947 |
| *CCDC136* | q32.1 | 0 | 0.000529 | 0.000529 | 0.000982 | 0.849775 | 0.900443 |
| *FAM200A* | q22.1 | 0 | 0.000256 | 0.000256 | 0.001063 | 0.845905 | 0.900443 |
| *LAMB1* | q31.1 | 0 | 0.005415 | 0.005415 | 0.025958 | 0.327165 | 0.551482 |
| *LRGUK* | q33 | 0 | 0.000152 | 0.000152 | 0.022906 | 0.364383 | 0.588087 |
| *MAGI2* | q21.11 | 0 | 0.004742 | 0.004742 | 0.044968 | 0.243954 | 0.481727 |
| *MBLAC1* | q22.1 | 0 | 0.000246 | 0.000246 | 0.012739 | 0.499882 | 0.698712 |
| *PCOLCE* | q22.1 | 0 | 0.002906 | 0.002906 | 0.013129 | 0.493345 | 0.692687 |
| *PODXL* | q32.3 | 0 | 0.007468 | 0.007468 | 0.053231 | 0.169488 | 0.379216 |
| *POLR2J2* | q22.1 | 0 | 0.000187 | 0.000187 | 0.000265 | 0.922788 | 0.942628 |
| *SEMA3A* | q21.11 | 0 | 0.004778 | 0.004778 | 0.06612 | 0.155392 | 0.356311 |
| *SMO* | q32.1 | 0 | 0.001197 | 0.001197 | 0.010138 | 0.541936 | 0.73044 |
| *ZC3HAV1L* | q34 | 0 | 0.00079 | 0.00079 | 0.009449 | 0.556069 | 0.743817 |
| *ZNF775* | q36.1 | 0 | 0.000157 | 0.000157 | 0.000268 | 0.916955 | 0.942628 |

**Table S10. List of the papers reporting lethality in knockout mice of the different 16 genes**

| **Gene** | **References** |
| --- | --- |
| *ALKBH4* | Li M. M.et al. (Nat Commun, 2009) |
| *CDK5* | Ohshima T. et al. (Proc Natl Acad Sci U S A, 1996) |
| *CEP41* | Lee J. E. et al. (Nat Genet, 2012) |
| *COPS6* | Zhao R. et al. (J Clin Invest, 2011) |
| *CUL1* | Nakagawa T. et al. (Adv Exp Med Biol, 2020) |
| *CUX1* | Luong M. X. et al. (Mol Cell Biol, 2002) |
| *DNAJC2* | Helary L. et al. (Biochem Biophys Res Commun, 2019) |
| *EZH2* | O'Carroll D. et al. (Mol Cell Biol, 2001) |
| *KMT2C* | Piunti A. et al. (Science, 2016) |
| *KRIT1* | Whitehead K. J. et al. (Development, 2004) |
| *RINT1* | Lin X. et al. (Mol Cell Biol, 2007) |
| *SSBP1* | Xu S. et al. (Cell Res, 2013) |
| *TRRAP* | Herceg Z. et al. (Nat Genet, 2001) |
| *WASL* | Lommel S. et al. (EMBO Rep, 2001) |
| *WDR91* | Liu K. et al. (J Cell Biol, 2017) |
| *XRCC2* | Deans B. et al. (EMBO J, 2000) |
